# Membrane extraction with styrene-maleic acid copolymer results in insulin receptor autophosphorylation in the absence of ligand

**DOI:** 10.1038/s41598-022-07606-5

**Published:** 2022-03-03

**Authors:** Kerrie A. Morrison, Laura Wood, Karen J. Edler, James Doutch, Gareth J. Price, Francoise Koumanov, Paul Whitley

**Affiliations:** 1 Department of Biology and Biochemistry, University of Bath, Bath, UK; 2 Department of Chemistry, University of Bath, Bath, UK; 3 Centre for Sustainable Circular Technologies, University of Bath, Bath, UK; 4 Department for Health, Centre for Nutrition, Exercise and Metabolism, University of Bath, Bath, UK; 5 ISIS Pulsed Neutron and Muon Source, Rutherford Appleton Laboratory, Harwell Oxford, Didcot, OX11 0QX UK; 6 Department of Chemistry, Khalifa University, Abu Dhabi, UAE

**Keywords:** Cell signalling, Growth factor signalling, Insulin signalling

## Abstract

Extraction of integral membrane proteins with poly(styrene-co-maleic acid) provides a promising alternative to detergent extraction. A major advantage of extraction using copolymers rather than detergent is the retention of the lipid bilayer around the proteins. Here we report the first functional investigation of the mammalian insulin receptor which was extracted from cell membranes using poly(styrene-co-maleic acid). We found that the copolymer efficiently extracted the insulin receptor from 3T3L1 fibroblast membranes. Surprisingly, activation of the insulin receptor and proximal downstream signalling was detected upon copolymer extraction even in the absence of insulin stimulation. Insulin receptor and IRS1 phosphorylations were above levels measured in the control extracts made with detergents. However, more distal signalling events in the insulin signalling cascade, such as the phosphorylation of Akt were not observed. Following copolymer extraction, in vitro addition of insulin had no further effect on insulin receptor or IRS1 phosphorylation. Therefore, under our experimental conditions, the insulin receptor is not functionally responsive to insulin. This study is the first to investigate receptor tyrosine kinases extracted from mammalian cells using a styrene-maleic acid copolymer and highlights the importance of thorough functional characterisation when using this method of protein extraction.

## Introduction

Poly(styrene-co-maleic acid) (SMA) copolymers have been shown to be a viable, versatile and potentially superior alternative to detergents for solubilising membrane components and facilitating the study of membrane proteins^1,2^. It is proposed that amphipathic SMA copolymers insert into membrane bilayers and ‘extract’ disc shaped particles that have been called ‘styrene maleic acid lipid particles’’ (SMALP’s) or ‘nanodiscs’. The particles have typical diameters of 10–30 nm and have a central segment of lipid bilayer surrounded by a copolymer ‘belt’^3,4^. The hydrophobic styrene moieties intercalate with the acyl chains of lipids and the hydrophilic maleic acid interacts with the aqueous environment and possibly the polar lipid head groups^5^.

Commercial copolymers with a 2:1 or 3:1 ratio of styrene:maleic acid, such as SMA2000, SMA3000 from Total Cray Valley (USA) and SZ30010 and SZ25010 from Polyscope (Netherlands), have been successfully used in the purification of a variety of membrane proteins^6–12^. However, these commercial copolymers are highly polydisperse with respect to monomer sequence and chain length. The development of copolymers synthesised using reversible addition-fragmentation chain transfer (RAFT) yielded materials with greater homogeneity^13,14^, controlled monomer sequence distributions and a narrow range of chain length. SMA copolymers are also sensitive to divalent metal cations and to neutralisation in buffers below pH6, effects which lead to nanodisc disassembly and precipitation respectively. This potentially limits the study of some membrane proteins^15,16^. To mitigate such limitations there has been a rapid development of other copolymers such as SMA-QA and diisobutylene/maleic acid (DIBMA) variants^17,18^.

Much of the characterisation of the physical properties of different copolymer lipid systems has been carried out using simple model membranes made up of defined compositions of lipids in the absence of proteins^8,12,14,19^. When extracting from biological membranes, copolymers can encapsulate proteins in addition to lipids. These protein/lipid polymer nanodiscs are proposed to be the minimal units of membrane protein function and given the name ‘memteins’^2^. Membrane proteins of many different types including ion channels^20^, and transporters^7^ have been successfully purified following extraction from membranes using SMA copolymers. Retention of functionality is more likely since proteins are present in a more natural lipidic environment in a memtein than when extracted from membranes using a detergent. However, there are relatively few examples where protein function in memteins has been addressed experimentally. This may be, at least in part, due to some functions of membrane proteins being difficult to assess in a nanodisc format. For example, measurement of ion or solute transport generally requires a sealed compartment, which is not provided by a nanodisc. However, inhibitor binding to transporters has been demonstrated, indicating a correctly folded conformation in the nanodisc^21^. There are however some elegant studies showing receptor proteins to be functional when incorporated into polymer nanodiscs. Ligand binding and subsequent recruitment of effectors has been demonstrated for different G-protein coupled receptors (GPCR’s) indicating function in a nanodisc format^22–24^. Studies on GPCR memteins have utilised heterologous recombinant expression and/or purification of engineered/tagged proteins in insect cells^25^, Pichia pastoris and HEK293 cells^22,26^, extraction with polymer and affinity purification prior to functional analysis.

The aim of this study was to assess the functionality of an important class of eukaryotic signalling receptors, receptor tyrosine kinases (RTK’s) when incorporated into SMA copolymer nanodiscs and the feasibility of in vitro reconstitution of downstream signalling events following receptor activation.

The insulin receptor (InsR) was chosen as a candidate RTK as it is a constitutive disulphide linked dimer (αβ)_2_^27^. RTK’s such as EGFR or PDGFR family receptors were not considered to be optimal for this initial ‘proof of concept’ study as they are normally monomeric and unlikely to be contained within the same nanodiscs following SMA extraction. EGFR and PDGFR rely on lateral movement within a membrane to become dimeric, a state which is only stabilised upon ligand binding^28^. As co-operativity between RTK subunits is a requirement for functionality, we concluded that the constitutively dimeric InsR is a rational choice for functional characterisation in a nanodisc format.

Activation of the InsR and downstream signalling proteins can be monitored by immunoblotting using phospho-specific antibodies as the active forms of the proteins are the phosphorylated forms. Upon insulin binding to the InsR extracellular domain, the intracellular domain of the receptor itself is first autophosphorylated at multiple tyrosine residues. This enhances InsR receptor tyrosine kinase activity and facilitates recruitment and phosphorylation of adapter proteins, such as insulin receptor substrate 1 or 2 (IRS1/2). This in turn leads to phosphoinositide 3-kinase (PI 3-kinase) activation and phosphorylation of Akt as well as MAPK pathway activation^29^.

The results reported here demonstrate that endogenous InsR can be extracted directly from cells by a SMA copolymer. However, extraction from basal cells, i.e. not treated with insulin, leads to unexpected InsR and IRS1 phosphorylation. Activation of the insulin signalling cascade downstream of IRS1 was not observed. The in vitro stimulation of SMA extracts with insulin did not result in activation of InsR. In this first study of an RTK in SMALPs we highlight findings that should be taken into consideration when employing copolymer technology to extract proteins from mammalian cells, in particular RTK’s.

## Results

The overall experimental strategy was to make SMA copolymer extracts from 3T3L1 fibroblasts that naturally express InsR. Subsequent stimulation of extracts with insulin and assessment of the phosphorylation status of proteins in the insulin signalling cascade would determine whether the InsR is functional when incorporated into the SMA nanodiscs. We reasoned that the extracts (soluble fractions) should contain both the InsR, if it is successfully incorporated into nanodiscs, and soluble downstream signalling components required for an in vitro assay.

### Proteins involved in insulin signaling cascade are present in the soluble fraction following SMA extraction

**Figure 1:**
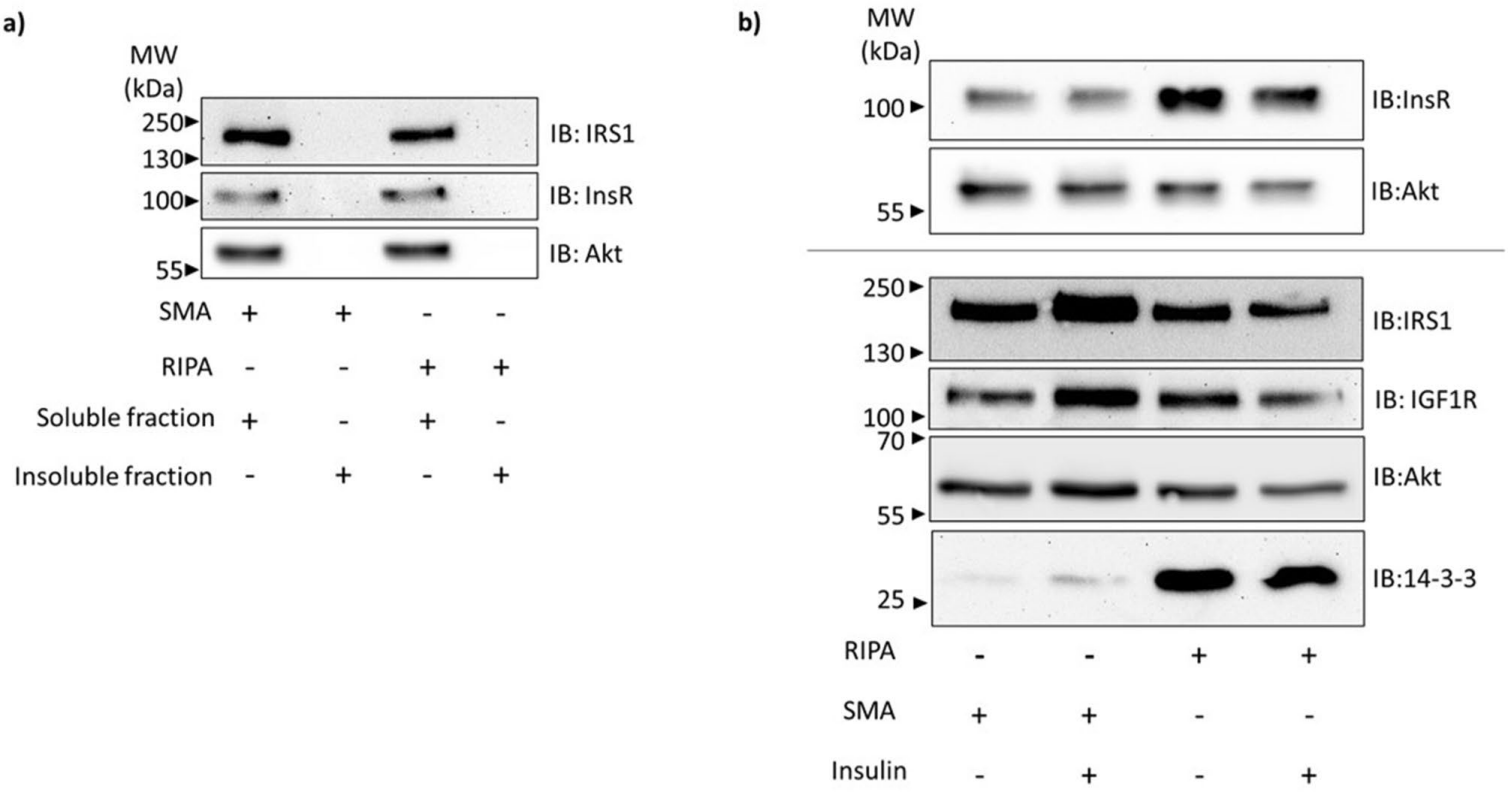
Fractionation of SMA and RIPA cell extracts to show the distribution of proteins involved in the insulin signalling cascade. (**a**) 3T3L1 cells were extracted with SMA or RIPA prior to ultracentrifugation for the preparation of soluble and insoluble fractions. (**b**) 3T3L1 cells were treated with insulin (+) or left untreated (−) prior to extraction with SMA or RIPA. Insoluble material was removed by centrifugation. Proteins in soluble fractions were separated by SDS-PAGE, transferred to nitrocellulose and detected by immunoblotting (IB) with antibodies specific to the indicated protein. Representative immunoblots are shown.

Firstly, we needed to establish whether the InsR and other proteins essential to the insulin signalling cascade can be efficiently extracted from mouse 3T3L1 fibroblasts using SMA. Cells were treated with SMA copolymer in PBS buffer containing no divalent cations at 37 °C. Cooling the cells prior to extraction is likely to result in artefactual lipid phase separations in membranes, potentially leading to proteins being extracted surrounded by non-native lipids. Insoluble material was removed from lysates by ultracentrifugation and the insoluble and soluble fractions analysed by Western blotting (Fig. 1a). Soluble fractions were prepared from cells that had been stimulated with insulin as well as from untreated (basal) cells (Fig. 1b). Treatment of cells with a detergent-containing radio-immunoprecipitation assay (RIPA) buffer was used as a control (Fig. 1b).

The results (Fig. 1a) demonstrate that extraction with SMA and RIPA resulted in essentially all the cellular InsR, IRS1 and Akt being in the soluble fractions following ultracentrifugation. These proteins are undetectable in the insoluble fraction. Our interpretation is that InsR, along with other integral membrane proteins including IGF1R, are extracted in nanodiscs in the SMA and detergent micelles in the RIPA soluble fractions, respectively. As expected, IRS1 and Akt are also recovered in the soluble fractions due to release of cell contents upon cell lysis with SMA or RIPA.

The addition of insulin to cells prior to extraction did not have any statistically significant effect on the extraction efficiency of InsR or IRS1 (Fig. 1b). We had intended to use 14-3-3 protein as an independent loading control. However, very little 14-3-3 protein is detected in the SMA soluble fraction compared with the RIPA soluble fraction. We had previously observed the same unexpected phenomenon for GAPDH, another cytoplasmic protein commonly used as a loading control (unpublished).

To further analyse the soluble fractions prepared following SMA and RIPA extraction, dynamic light scattering (DLS) was performed (Supplementary material, Fig. S1). As the fractions contain soluble membrane proteins and cytoplasmic/organellar components released upon cell lysis it was expected that the samples would contain a complex mixture of different sized particles. Thus, the results are difficult to interpret with confidence. A high polydispersity index (PDI) of 0.66 was observed in the SMA-soluble fraction, while the RIPA-soluble fraction had a lower PDI of 0.26. The data presented by intensity show the presence of three peaks in both samples. For the SMA extracted fraction the first peak occurs at a diameter of 16 nm, while in the RIPA extracted fraction it is found at 5 nm. This is consistent with particles present in the SMA soluble fraction being nanodiscs and those in the RIPA soluble fraction being micelles. However, the calculated volume data has a single peak in both samples, at smaller diameters, suggesting that the predominant material in both samples consists of the co-extracted soluble proteins, which are expected to be around 2–8 nm in diameter. This distribution is notably skewed in the SMA containing fraction, presumably due to the presence of the nanodiscs. The other peaks, seen only in the intensity data, occur at roughly the same diameter in both samples and are attributed to small amounts of soluble protein aggregates co-extracted in both cases.

### Extraction with SMA from unstimulated cells leads to activation of the insulin receptor

**Figure 2:**
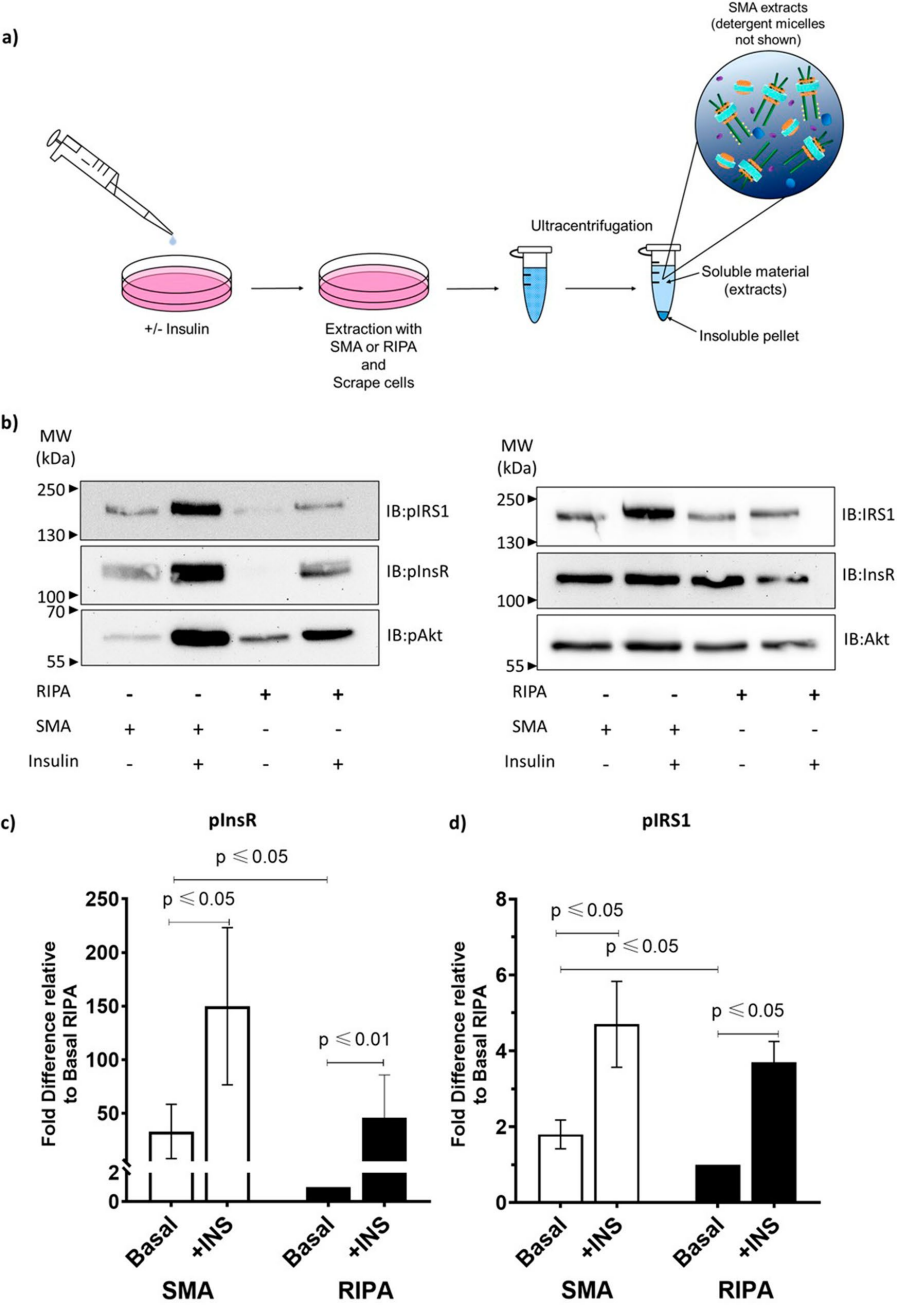
Analysis of activation status of insulin signalling cascade proteins in SMA and RIPA soluble fractions. (**a**) Schematic diagram of the experimental protocol for making extracts from 3T3L fibroblasts. Cells were treated with 100 nM insulin for 30 min (+Insulin) or left untreated (−Insulin) prior to extraction with SMA or RIPA. Insoluble material was removed by centrifugation and proteins were analysed by immunoblotting (IB) with specific antibodies (**b**) Representative immunoblots are shown with phospho-specific antibody detection (left-panel) and corresponding total protein (right panel). (**c**) Quantification of InsR phosphorylation levels is expressed as a ratio of pInsR/total InsR and compared to the ratio from basal cells extracted with RIPA ‘fold difference relative to Basal RIPA’. (**d**) Quantification of IRS1 phosphorylation levels expressed as a ratio of pIRS1/total IRS1 and compared to the ratio from basal cells extracted with RIPA ‘fold difference relative to Basal RIPA’. Values are means ± SEM, *n* = 3 (InsR) and *n* = 4 (IRS). *P*-values were determined by paired t-test. Additional material: Specificity of phospho-specific antibodies (Supplementary Material Fig. S2). Enhanced contrast image of immunoblot (left panel, row 2) showing basal pInsR levels in untreated RIPA extracts (−Insulin) (Supplementary Material Fig. S3).

Next, we investigated the activation status of the insulin signalling cascade proteins in the soluble fractions. Soluble fractions were prepared from unstimulated (basal) or insulin treated cells extracted with SMA or RIPA (Fig. 2a). The insulin treated cells should contain the activated (phosphorylated) forms of InsR, IRS1 and Akt. Phosphorylation status was assessed using phospho-specific antibodies (Supplementary material Fig. S2).

As expected, SMA and RIPA soluble fractions from cells stimulated with insulin had increased levels of pInsR, pIRS1 and pAkt (Fig. 2b, left panel). Surprisingly, in unstimulated cells extracted with SMA the levels of pInsR and pIRS1 were significantly higher than the levels detected upon RIPA extraction (Fig. 2b left panel, Fig. 2c and d). It should be noted, that the pInsR levels observed following SMA extraction of unstimulated cells were significantly lower (two-tailed paired t-test, *p* = 0.017) than those from insulin stimulated cells, suggesting there was still capacity for more phosphorylation and activation of the InsR-nanodiscs. Additionally, downstream activation of Akt above basal levels is not observed upon SMA extraction from unstimulated cells, whereas it is clearly activated in insulin stimulated cells (Fig. 2b, left panel). Although the intensity of the pInsR band appears greater in SMA than RIPA soluble fractions from insulin stimulated cells (Fig. 2b), upon quantification, this difference was not found to be statistically significant (*p* = 0.052) (Fig. 2c).

**Figure 3:**
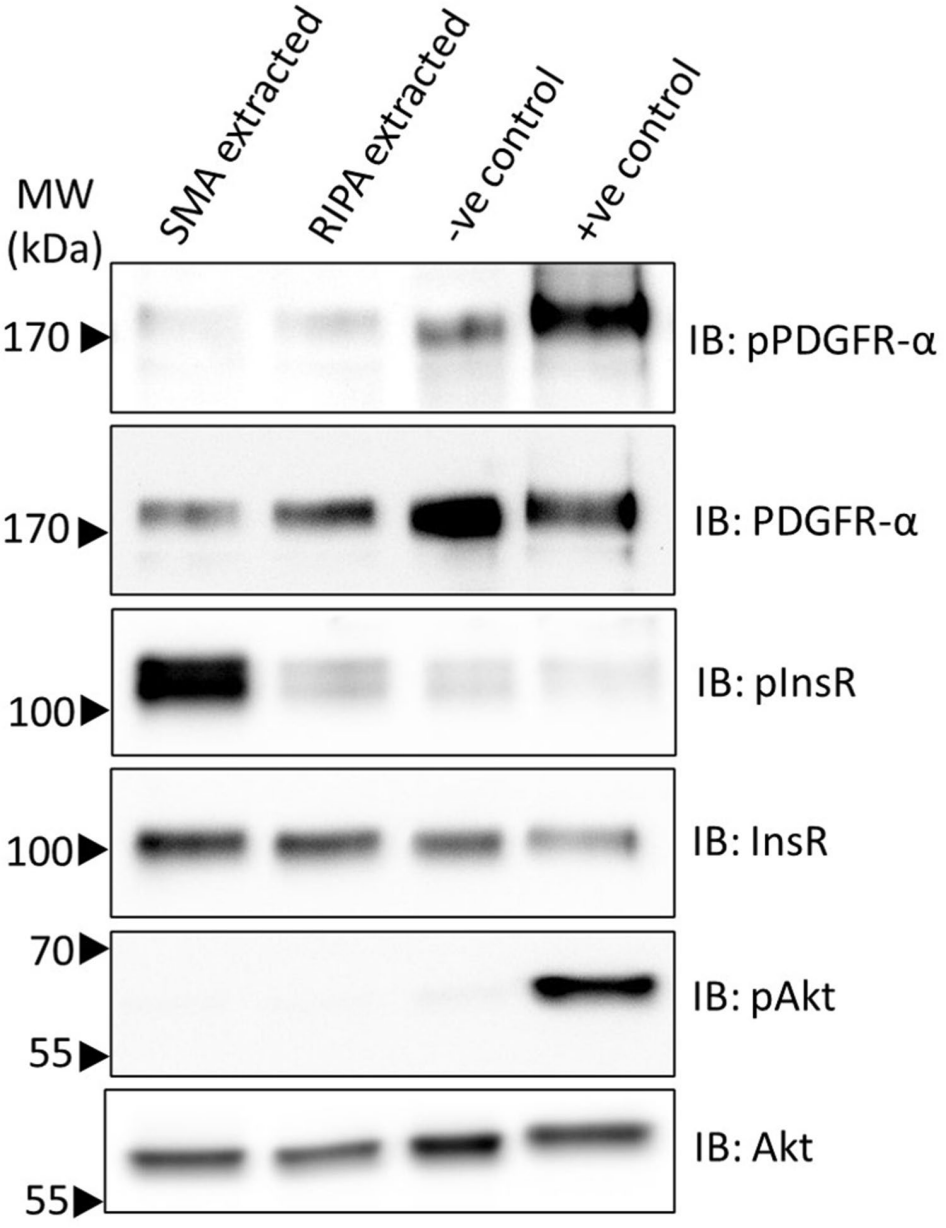
Analysis of activation status of PDGFRα proteins in SMA and RIPA soluble fractions. Untreated 3T3L1 fibroblasts were extracted with SMA or RIPA. Insoluble material was removed by centrifugation and proteins were analysed by immunoblotting (IB) with specific antibodies. As controls, samples from untreated cells (−ve control) and cells treated with PDGF-BB ligand (+ve control) were prepared and separated on the same gel.

To investigate whether other RTKs became phosphorylated upon extraction with SMA from unstimulated cells, immunoblotting for phosphorylated PDGFRα was performed (Fig. 3).

There was no significant difference (*p* = 0.55) in the phosphorylation status of PDGFRα upon extraction with SMA compared to RIPA (Supplementary Fig. S4). PDGFRα is phosphorylated upon addition of PDGF-BB ligand to cells, demonstrating that phosphorylated PDGFRα can be detected (Fig. 3).

### In vitro activation of the InsR memteins is not detected upon addition of insulin.

**Figure 4:**
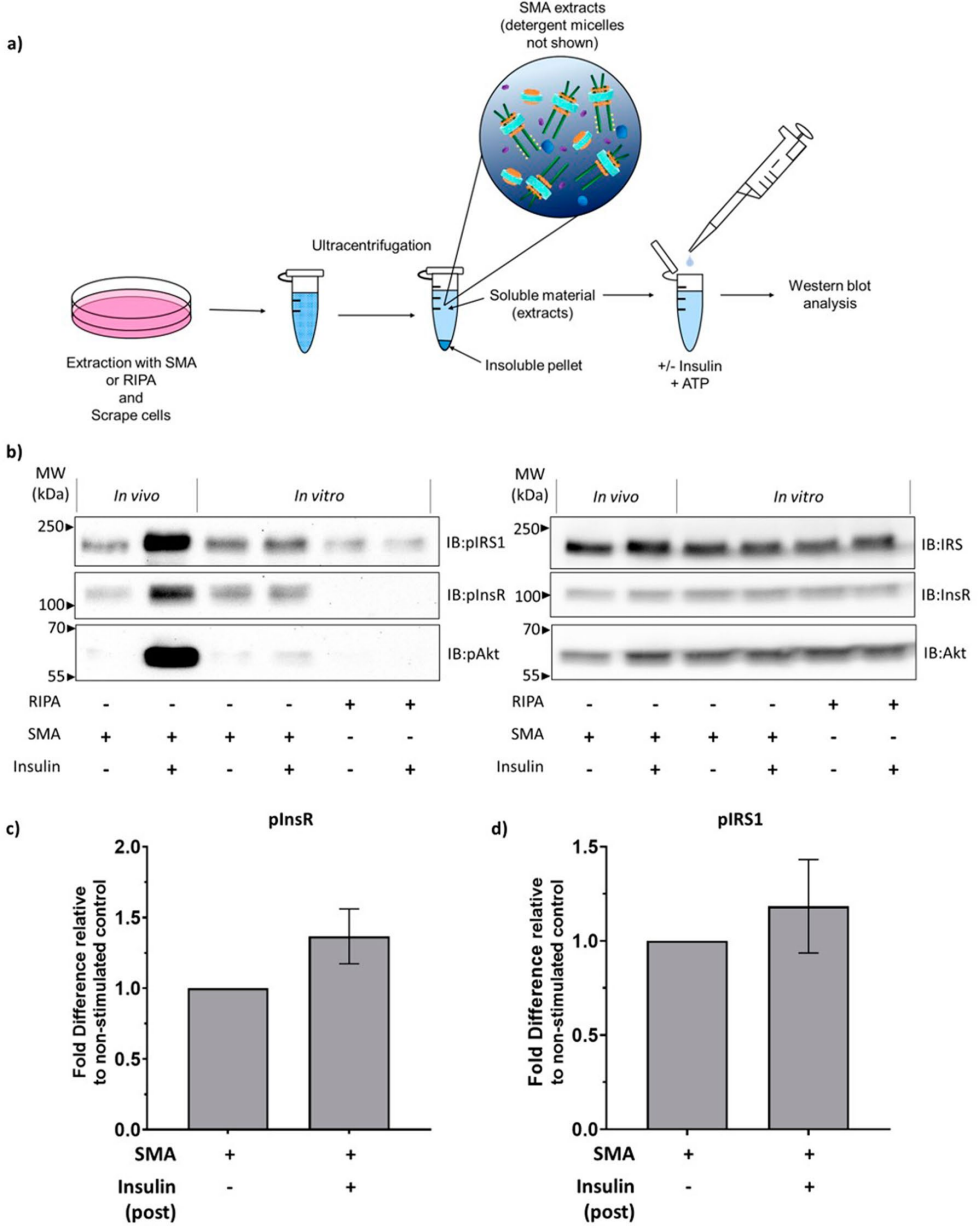
Analysis of in vitro activation of InsR by insulin following extraction from cells. (**a**) Schematic diagram of the experimental protocol for in vitro stimulation of SMA/RIPA extracts. Untreated cells were extracted with SMA or RIPA and insoluble material was removed by centrifugation. Extracts were treated with 100 nM insulin (in vitro) (+Insulin) or left untreated (−Insulin). (**b**) Representative immunoblots are shown with phospho-specific antibody detection (left-panel) and corresponding total protein (right panel). Extracts from cells treated with 100 nM insulin (+Insulin) or untreated (−Insulin) prior to extraction (in vivo) with SMA are also shown on the same gel for comparison. (**c**) Quantification of InsR phosphorylation levels in SMA extracts. Data are expressed as ratio of pInsR/total InsR and compared to the ratio from basal cells extracted with SMA as fold difference relative to non-stimulated control. Values are means ± SEM, *n* = 3. *P*-values were determined by paired t-test. (**d**) Quantification of IRS1 phosphorylation levels in SMA extracts Shown as ratio of pIRS1/total IRS1 and compared to the ratio from basal cells extracted with SMA as fold difference relative to non-stimulated control. Values are means ± SEM, *n* = 3. *P*-values were determined by paired t-test.

The initial aim of this study was to investigate the functional state of SMA extracted InsR and whether the insulin signalling cascade could be initiated in vitro by addition of insulin to SMA extracted soluble fractions (post-SMA). However, the unexpected result that InsR was significantly phosphorylated above basal levels, when extracted with SMA, was a confounding factor to this plan. Despite this, the pInsR levels observed following SMA extraction of unstimulated cells were significantly lower than those from insulin stimulated cells, we considered that there was still capacity for more phosphorylation and activation of the InsR-nanodiscs. Therefore, we performed an in vitro experiment to test if insulin addition to SMA or RIPA soluble fractions post extraction from untreated cells would stimulate the signalling cascade (Fig. 4a).

The results presented in Fig. 4b show that addition of insulin in vitro to SMA extracted soluble fractions containing InsR memteins led to a small but statistically insignificant increase in pInsR levels compared to untreated samples (Fig. 4c). The same was observed for pIRS1 levels (Fig. 4d). InsR and IRS1 in RIPA extracts did not become phosphorylated above background levels when incubated with insulin. No effect on Akt phosphorylation was observed upon insulin addition to SMA or RIPA cell extracts.

## Discussion

The experiments described in this study reveal that the insulin receptor can be successfully extracted from intact cells using a SMA copolymer. We reason that the insulin receptor present in the soluble fraction has been incorporated into nanodiscs. The presence of nanodiscs is supported by the DLS which indicates the detection of 16 nm particles in the SMA extracted samples that are absent from the RIPA extracted samples. We must, however, interpret the DLS data with caution due to the heterogeneous nature of the extracts which contain solubilised integral membrane proteins and soluble proteins released upon cell lysis.

Unexpectedly, we observed an induction of InsR phosphorylation when extracts are made with a SMA copolymer from basal cells, i.e. cells that have not been treated with insulin. This InsR phosphorylation induced by SMA extraction, is presumably an autophosphorylation event between cytoplasmic tyrosine kinase domains, which is usually controlled by the binding of insulin to extracellular domains of the receptor. The most likely explanation is that extraction of the constitutively dimeric InsR into SMA nanodiscs results in conformational changes that lead to autophosphorylation even in the absence of insulin. This is supported by our observation that PDGFRα, which is a monomeric RTK, does not become phosphorylated upon SMA extraction from unstimulated cells. Extraction with SMA should result in monomers that are distant from one another in membranes being incorporated into separate nanodiscs making it impossible for kinase domains to become juxtaposed and cross (auto) phosphorylate. We predict that this monomeric status also excludes it being suitable for in vitro stimulation experiments once incorporated into nanodiscs. It is interesting to note that reconstitution of purified recombinant InsR into membrane scaffold protein (MSP) bound nanodiscs has also been reported to result in InsR phosphorylation without addition of insulin^30^. In both our study and when the InsR is incorporated into MSP nanodiscs it is only a sub-population of the InsR that becomes phosphorylated. We deduce this since InsR extracted with SMA from insulin stimulated cells has a much higher level of phosphorylation (4.7-fold higher) than InsR extracted from untreated cells (Fig. 2). As the total amount of InsR extracted is the same in both conditions it is only the proportion that is phosphorylated that is different. In MSP nanodiscs, the proportion of InsR that was phosphorylated increased upon the addition of insulin and ATP^30^. It should be noted that while relative amounts of phosphorylation between conditions can be determined, the percentage of total InsR that is phosphorylated cannot be measured using immunoblotting.

A novel finding of our study is that pIRS1 levels are also above basal levels in soluble fractions following extraction with SMA from unstimulated cells. We propose that the pool of pInsR, induced upon SMA extraction, recruits and phosphorylates IRS1. This mimics the early steps of the insulin signalling cascade and would indicate that some InsR at least is functionally active, during (or following) the SMA extraction procedure. However, phosphorylation of Akt above basal levels is not observed upon SMA extraction from cells. Thus, while there is phosphorylation of InsR and IRS1 upon SMA extraction, the full insulin signalling pathway activation is not achieved. Akt activation requires PI 3-kinase to be recruited to the activated InsR, production of phosphatidylinositol (3,4,5) trisphosphate from phosphatidylinositol (4,5) bisphosphate and association of PDK1, mTORC2 and Akt to the same area of membrane^34,35^. The available surface area of an individual InsR containing nanodiscmay impose spatial constraints to reconstituting such signalling pathways in vitro from receptors incorporated into nanodiscs. While able to facilitate InsR phosphorylation and IRS1 recruitment, nanodiscs may be too small to accommodate all the subsequent events required for pathway activation.

The initial aim of this study was to assess the functionality of the InsR when incorporated into SMA nanodiscs. It has previously been shown that InsR incorporated into MSP nanodiscs are seemingly functional, as insulin and ATP addition induced InsR phosphorylation above levels observed in untreated nanodiscs. Additionally, large conformational changes in the InsR, visualised by cryo-EM, were observed, with two previously separated kinase domains being brought into juxtaposition^30^. Unlike the aforementioned study, which consisted of purified recombinant InsR incorporated into MSP’s, our in vitro assay consisted of a heterogeneous mixture of integral membrane proteins including InsR, presumably in SMA nanodiscs, and soluble proteins. While lacking a well-defined constitution, our experimental approach should, in theory be more representative of a cellular environment and allow monitoring of downstream signalling from the insulin receptor. However, we only observed a statistically insignificant 1.4-fold increase in pInsR levels when insulin and ATP was added to SMA extracts. A very similar observation was made for pIRS1 levels. Thus, we have no evidence that the InsR incorporated into SMA nanodiscs is in a functional state, able to initiate a signalling cascade upon insulin addition. A potential reason for this is that the SMA copolymer makes unfavourable interactions with InsR extracellular or kinase domains making it unresponsive to insulin. Of note is the study by Gutmann et al.^30^ who reported that 80% of the recombinant InsR used in their experiments reconstituted in double nanodiscs (each TM in a different nanodisc), preventing the juxtaposition of the two transmembrane domains of the receptor upon insulin stimulation in vitro. Despite the choice of InsR to study because it is constitutively dimeric, we cannot rule out that this is not also the case in SMA nanodiscs, preventing a further activation of the InsR in vitro. Further experimentation, by for example alteration of kinase assay conditions or increasing the concentrations of insulin to 1 μM^30^ may be necessary before we can categorically conclude that InsR incorporated into SMA nanodiscs is not functional. It is also possible that screening of polymers with different chemistries^18,36–42^ may be necessary to facilitate extraction of InsR and/or other RTK’s in a form more favourable to in vitro functional studies.

While not directly related to the primary aims of the project, an observation was made when making extracts from mammalian cells, that cytoplasmic proteins such as 14-3-3 are abundant in RIPA extracts yet were barely detectable in SMA extracts. We have observed the same phenomenon for GAPDH in other studies (unpublished). When extracting from intact cells using SMA, the expectation was that all cytoplasmic proteins will be released into the solubilisation buffer along with the membrane proteins incorporated into nanodiscs. Cytoplasmic proteins and memteins should remain in the soluble fractions following centrifugation. The lack of detection, by immunoblotting, of some proteins such as 14-3-3 and GAPDH but not others such as Akt or IRS1 requires further investigation. It could be that the presence of SMA copolymer leads to aggregation of some proteins, or the polymer could interact unfavourably and interfere with the separation of certain proteins on SDS-PAGE. Whatever the explanation, this selective apparent depletion needs to be taken into consideration when selecting loading controls for immunoblotting experiments.

In conclusion, this study has demonstrated that we are able to efficiently extract membrane receptors of the RTK family from cellular membranes using SMA. However, this study has also brought to light some of the issues that may be encountered when attempting to investigate functionality of membrane proteins extracted from mammalian cells. These issues are potentially due to changes in the protein environment, such as minor conformational changes brought about by alterations in lateral membrane pressure when incorporated into a nanodisc as InsR phosphorylation is also observed upon incorporation into MSP nanodiscs^30^. Another possibility is that unfavourable interactions between polymer and protein result in loss of function. It cannot be assumed that because integral membrane proteins can be extracted using methodology likely to retain their natural lipidic environment that they will remain in a fully functional conformational state. Activity needs to be determined on a case-by-case basis.

## Methods

### Materials

SMA2000 (a copolymer of styrene and maleic anhydride) was supplied by Cray Valley and hydrolysed to form SMA using the protocol described by^37^. Insulin, Zinc, Human, Recombinant, P. pastoris (#407709), Adenosine 5’-triphosphate magnesium salt from bacterial source (Mg-ATP) (#A9187) and ECLTM Select Western Blotting Detection Reagent (#GERPN2235) was supplied by Sigma Aldrich. NC Nitrocellulose Membranes (#15249794, Cytiva Amersham™ Protran™). Gibco™ 10 × concentrated Dulbecco’s phosphate-buffered saline (#14200-067), HaltTM Protease & Phosphatase Single-use Inhibitor Cocktail (100x) (#78442) and HaltTM Protease Single-use Inhibitor Cocktail (100x) (#78430) were supplied by ThermoFisher Scientific. Benzonase® Nuclease HC, Purity > 90% (#71205, Millipore). Calf intestine alkaline phosphatase Quick CIP (#M0525, New England BioLabs). Unless otherwise stated all chemical reagents and cell culture media and supplement were from Sigma Aldrich.

### Antibodies

Anti Insulin receptor β (4B8) (#3025)(1:1000 working dilution); Anti IGF-1 Receptor β (#3027) (1:1000); Anti Akt2 (D6G4) (#3063) (1:1000); Anti phospho-IGF-1 Receptor β (Tyr1135/1136) / Insulin Receptor β (Tyr1150/1151) (19H7) (1:500); Anti Phospho-Akt (Ser473) (193H12) (#4058) (1:1000); Anti phospho-PDGFR-α (Tyr754) (#2992) (1:1000); Anti PDGFR-α (#3174) (1:1000) were supplied by Cell Signalling Technology^®^. Pan 14-3-3 (K-19) (#sc-629) (1:1000) was from Santa Cruz Biotechnology Inc. Anti phospho-IRS1 (Tyr 608) (#09-432) (1:1000) and anti IRS1 (#06-248) (1:500) were supplied by Millipore™. Secondary antibody; Goat anti-Rabbit IgG (H + L) Cross-Adsorbed Secondary Antibody, HRP (#A16104) (1:4000) was supplied by Invitrogen.

### Cell culture

3T3-L1 fibroblast were purchased from American Tissue Culture Collection and were cultured in Dulbecco’s Modified Eagle’s Medium—high glucose (DMEM, supplemented with 10% Newborn Bovine Serum, 2 mM glutamine, and 100 U/ml penicillin/ 100 ug/ml streptomycin^43^) up to ~ 70–80% confluency before splitting. 3T3L1 mouse fibroblasts were grown to ~ 90% confluency in 10 cm diameter cell culture dishes (approx. 10^7^ cells) for experimentation (Corning, Fisher Scientific).

### Insulin stimulation of cells and preparation of cell extracts

Prior to experiments cells were washed in Gibco™ Dulbecco’s phosphate-buffered saline (#14200-067) and incubated in serum-free DMEM for 2 h. For experiments in which cells were incubated with insulin before extraction with SMA or RIPA (pre extraction), they were incubated with serum free DMEM containing 100 nM insulin for 30 min at 37 °C^44^. The cells were then washed in PBS (12.5 mM Sodium phosphate dibasic dodecahydrate 154 mM NaCl, pH 7.4), before extraction using either 1.5 wt. % SMA in PBS or RIPA buffer (1% Triton X-100, 0.2% SDS, 1% Sodium Deoxycholate in PBS), 500 µL per 10 cm dish. Buffers were supplemented with 1 × protease & phosphatase Single-use inhibitor Cocktail. Cells were scraped from the plates and incubated for 1 h at 37 °C, under mild agitation to ensure maximal extraction. 75 units of Benzonase® Nuclease was added to each sample for 5 min at 37 °C to digest DNA and reduce viscosity. Insoluble components were removed through ultracentrifugation at 170,000 × g (Optima Max ultracentrifuge with TLA100.3 rotor, Beckman Coulter) for 20 min at 25 °C. The supernatants which are the soluble fractions, was removed from the insoluble pellets.

### Post extraction insulin stimulation of soluble fractions

100 nM insulin and 100 µM Mg-ATP was added to the soluble fractions and incubated for 30 min at 37 °C under mild agitation.

### SDS PAGE and Immunoblotting

Samples were prepared in SDS-sample buffer containing 20 mM dithiothreitol (DTT) and heated for 5 min at 95 °C. Typically, 18 µg of protein was added per lane and separated on 10% SDS polyacrylamide gels and transferred onto nitrocellulose using a semi-dry transfer apparatus. Ponceau S stained nitrocellulose membrane blots were cut between the appropriate molecular weight markers prior to immunoblotting with specific antibodies. Blocking was in Tris-buffered saline (TBS, 0.9% NaCl, 10 mM Tris–HCl, pH 7.4), 0.1% Tween 20, 5% non-fat skimmed milk powder (Marvel) or TBS 0.1% Tween 20, 1% bovine serum albumin (BSA) (for phospho-specific antibodies). Primary antibodies were diluted (see above for dilutions) in TBS, 0.1% Tween 20, 1% BSA. Secondary antibody was diluted in TBS, 0.1% Tween 20, 5% Marvel. All washes were in TBS, 0.2% Tween 20. ECL™ Select Western blotting detection reagent was used for detection. Images were acquired with EPI Chemi II darkroom (UVP). Protein concentrations in samples were determined using BCA Protein Assay Kit, ThermoFisher Scientific, following the manufacturers protocol. Quantification of bands on immunoblots was measured by densitometry using Image Studio Lite (LI-COR Biosciences^®^). Specific band intensities were normalised to total Akt levels for that sample. The ratios between phospho-specific and total protein band intensities were calculated in the same sample. Results are presented graphically as fold-difference relative to control as indicated in the figure legend. Uncropped images are included in the supplementary file.

### Statistics

Results are presented as mean ± SEM unless otherwise stated. Two-tailed paired t-tests were used to determine the statistical significance (*p* value < 0.05) of phospho:total protein band intensity ratios between different conditions. A minimum of 3 independent experiments were used for pairwise comparisons.

## Supplementary Information

Supplementary Information.

## Data Availability

Supporting information is freely available for download in the Bath Research Data Archive; https://doi.org/10.15125/BATH-01041.
