# Supplementary material for: Membrane extraction with styrene-maleic acid copolymer results in insulin receptor autophosphorylation in the absence of ligand
Source: Sci Rep. 2022 Mar 3;12:3532. doi: 10.1038/s41598-022-07606-5 (PMC8894449; doi:10.1038/s41598-022-07606-5)
Supplement: Supplementary file 1 — Supplementary Information. [file 41598_2022_7606_MOESM1_ESM.docx]

**Supplementary Figures**

# Dynamic Light Scattering (DLS) Sample preparation method

DLS experiments were carried out at 20 °C on a Malvern Panalytical Zetasizer Nano ZS, at a wavelength of 633 nm and an angle of 173°. 40 μl plastic cuvettes were used for each sample. Samples were diluted to 0.1 mg/mL (diluted 5x) in PBS.


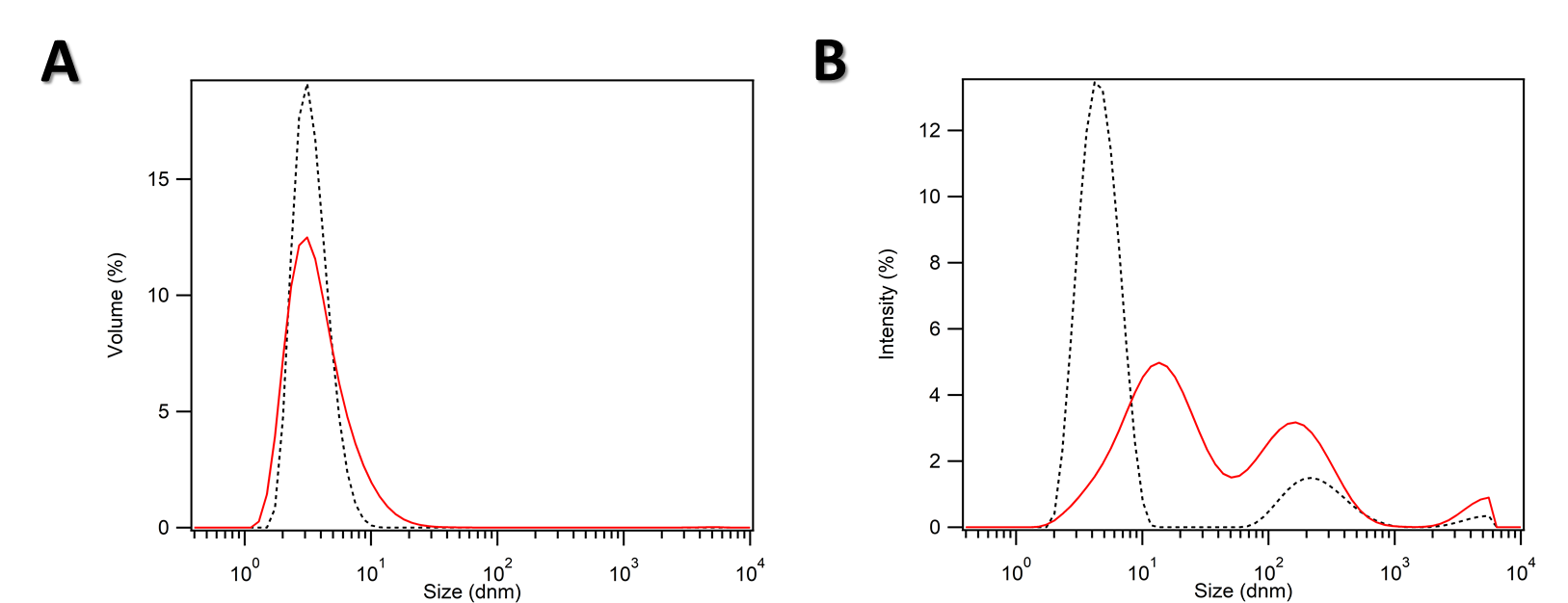


Figure S1. Comparison for SMA and RIPA soluble fraction after centrifugation by DLS. (A) Particle size distributions (nm) by volume (%) are expressed as a function of particle diameter (dnm). (B) Particle size distributions (nm) by intensity (%) are expressed as a function of particle diameter (dnm). (Red) correspond to the SMA-solubilised sample after removal of insoluble extracts using centrifugation. (Black, dotted) correspond to the RIPA-solubilised equivalent. The Z-average diameter in the SMA soluble fraction has a PDI of 0.66 while in the RIPA buffer it has a PDI of 0.27. From the first peak in the particle size distribution by intensity %, the SMALP diameter was calculated at 16 nm in diameter while the smallest aggregate in the RIPA buffer was 5 nm in diameter, close to the expected size of the detergent micelles.

# Phosphatase treatment of samples

To be certain that the protein signals detected by the phospho-specific antibodies were due to phosphorylation and not artefactual, soluble fractions were treated with phosphatase (Quick CIP) prior to SDS PAGE and western blotting (Supplementary material Fig.S2). Samples were prepared in the same manner as described in Method section (Section 2.4 and 2.5), with the exception that phosphatase inhibitors were excluded from the SMA and RIPA extraction buffers. Quick CIP was added to the centrifuged soluble fractions at a concentration of 1 unit per µg of protein. Samples were incubated under mild agitation for 1 hour at 37°C. The results show that bands detected by phospho-specific antibodies were much reduced in phosphatase treated samples confirming that the anti pInsR, anti pIRS1 and anti pAkt antibodies recognise phosphorylated versions of their respective targets and not unphosphorylated target.


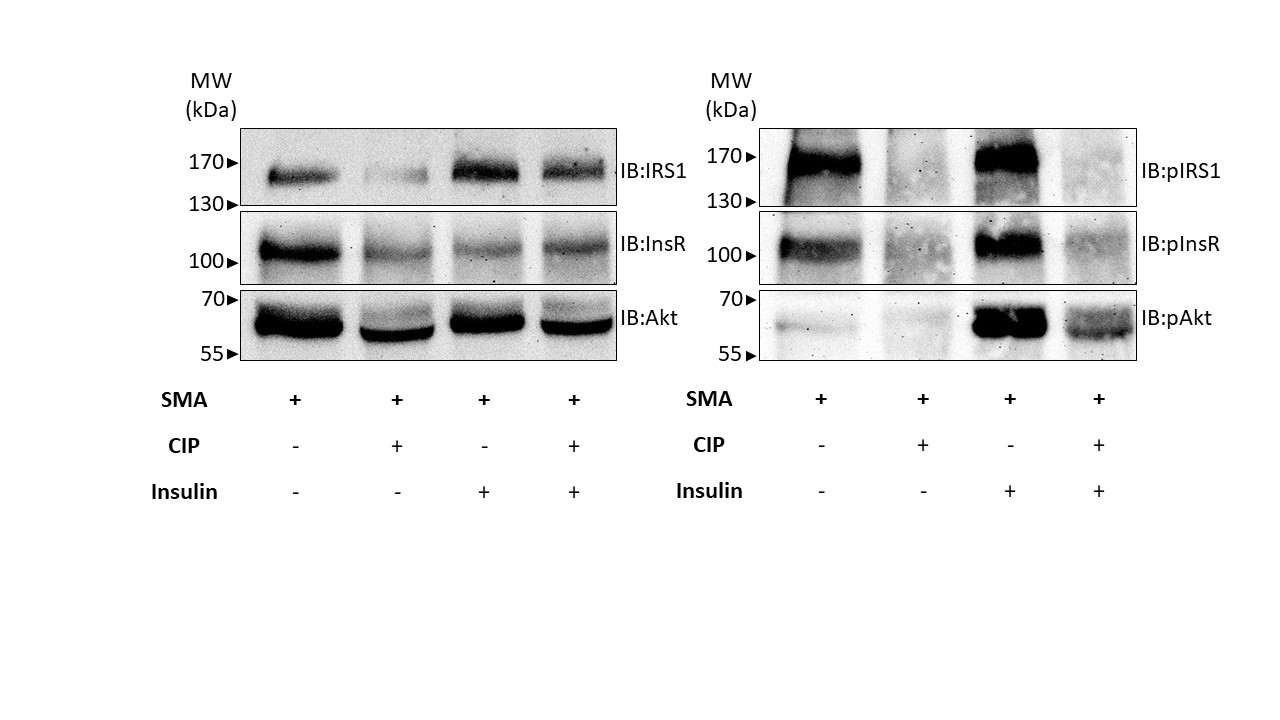


Figure S2. Samples were treated or untreated with insulin prior to extraction with SMA. Samples were treated with CIP or left untreated. Representative immunoblots are shown.


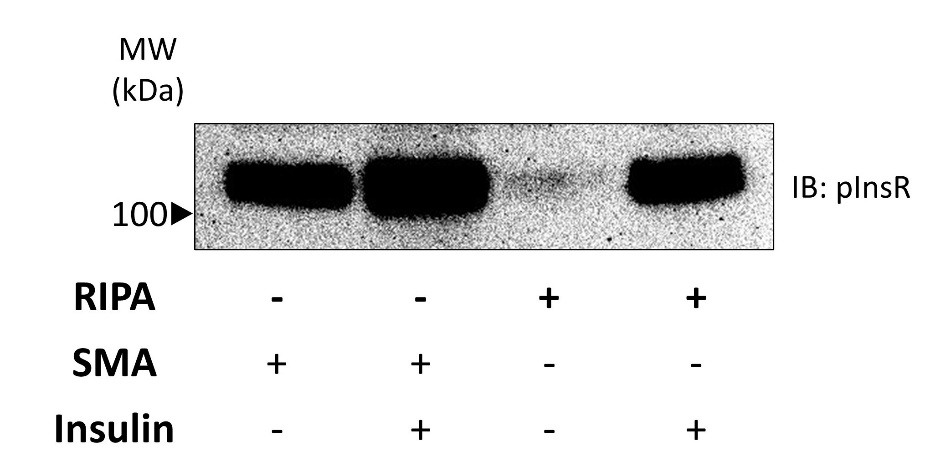


Figure S3. Enhanced contrast pInsR immunoblot from Figure 2B (left panel, row 2). This shows basal pInsR levels in untreated RIPA extracts detected from the pInsR phospho-specific antibody (lane 3).


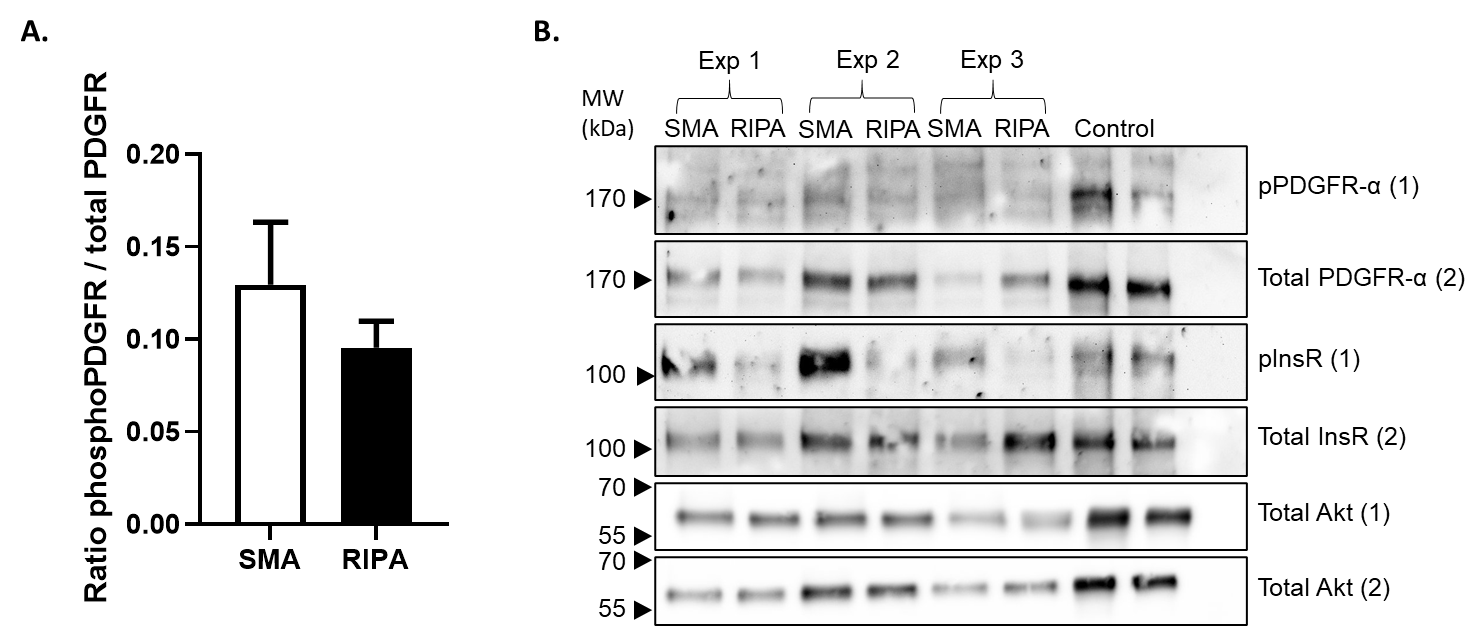


**Figure S4.** Analysis of activation status of PDGFRα proteins in SMA and RIPA soluble fractions from 3T3L1 fibroblasts. Untreated cells were extracted with SMA or RIPA as described in the Method section of the main manuscript. Extracts from 3 independent experiments were loaded on the gel as indicated (Exp 1 to 3). As a positive control, cells were left untreated or treated with 30ng/ml PDGF-BB ligand (PeproTech, #315-18) for 5 min at 37^o^C prior to lysis with SDS sample buffer. Insoluble material was removed from all samples by centrifugation and proteins were analysed by immunoblotting (IB) with specific antibodies. PDGFR-α activation was observed using phospho-PDGFR-α (Tyr754) antibody (Cell Signalling Technologies, #2992) and compared to total PDGFR-α antibody (Cell Signalling Technologies, #3174). **A.** Quantification of the ratio of phospho PDGFRα / total PDGFRα for SMA and RIPA extracted samples. Data are Mean +/- SEM (n=3). Comparison SMA to RIPA p= 0.55 (non-significant) using paired two-tailed Student’s T-test. **B**. Western blot images used for the quantification. To validate the experiments membranes were also probed antibodies for phospho InsR, total InsR and Akt to match with the results reported in figure 2 of the main article.

**Complete jpg images of the immunoblots presented in Figures 1, 2, 3 and 4 in the main manuscript and Supplementary figures S2, S3 and S4. The raw image TIFF files are available in the Bath Research Data Archive; DOI: https://doi.org/10.15125/BATH-01041.**

**Green coloured rectangles indicate the area which was used for the representative images in the main text figures.**

**Figure 1a**


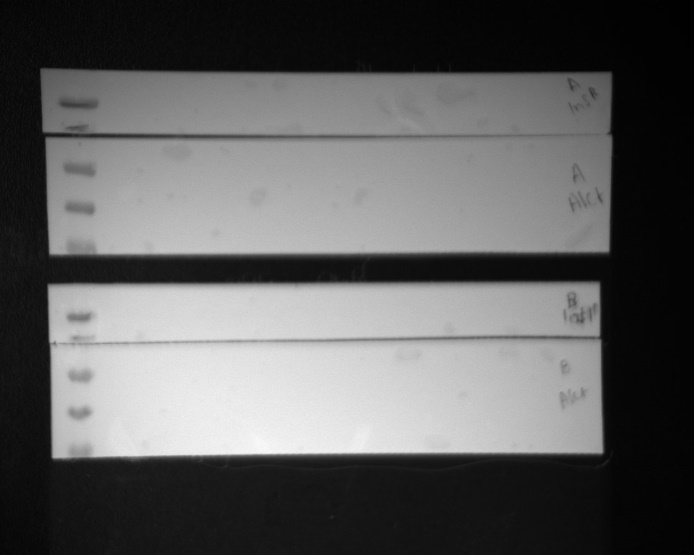


100

70

55

40

35

100

70

55

40

35

Markers image for Akt and InsR blots

MW

(kDa)


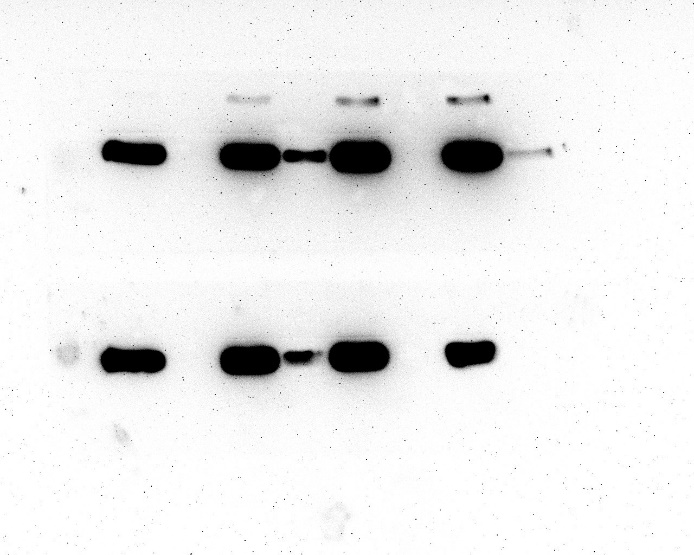

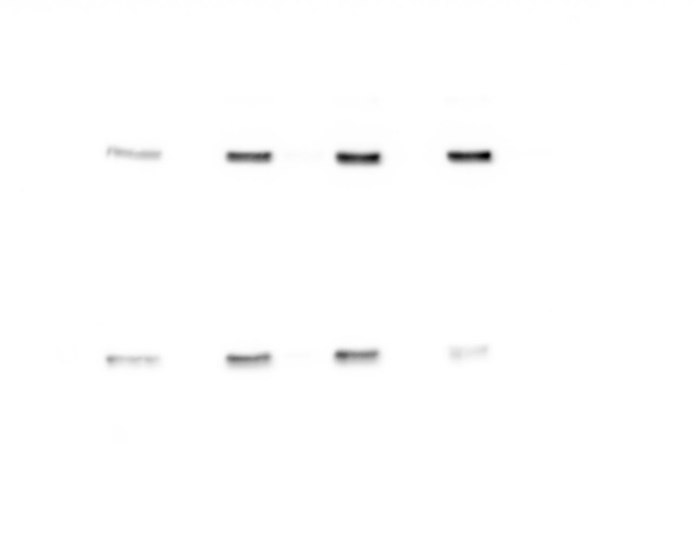


100

70

55

40

35

100

70

55

40

35

100

70

55

40

35

100

70

55

40

35

MW

(kDa)

MW

(kDa)

Akt blot (short exposure)

InsR blot (long exposure)

RIPA - - + +

SMA + + - -

Soluble + - + -

Pellet - + - +

RIPA - - + +

SMA + + - -

Soluble + - + -

Pellet - + - +

RIPA - - + +

SMA + + - -

Soluble + - + -

Pellet - + - +

**Figure 1a (continue)**


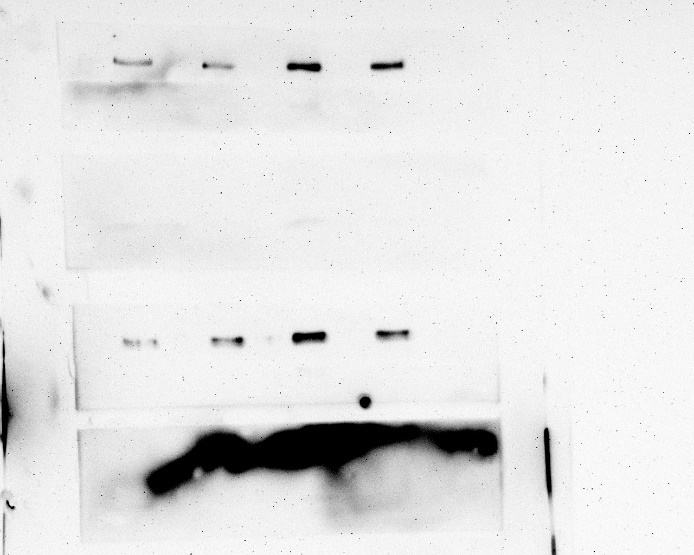

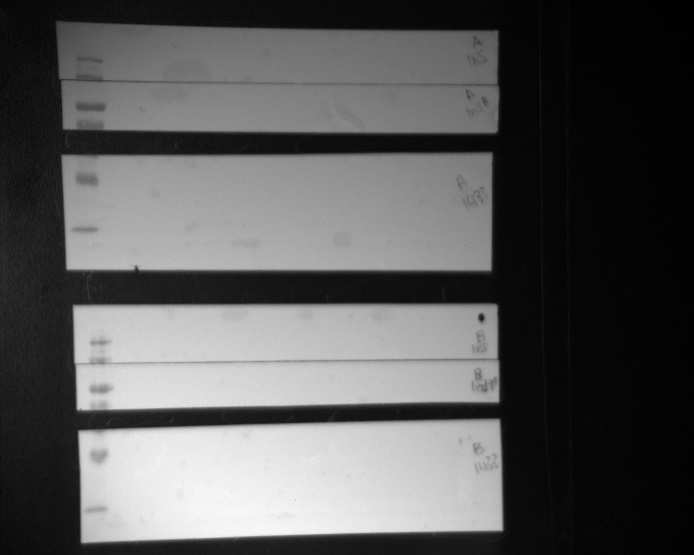


100

70

180

40

35

25

130

100

70

180

40

35

25

130

100

70

180

40

35

25

130

100

70

180

40

35

25

130

Markers image blot for IRS1 blot

IRS1 blot (long exposure)

MW

(kDa)

MW

(kDa)

RIPA - - + +

SMA + + - -

Soluble + - + -

Pellet - + - +

RIPA - - + +

SMA + + - -

Soluble + - + -

Pellet - + - +

**Figure 1 b**


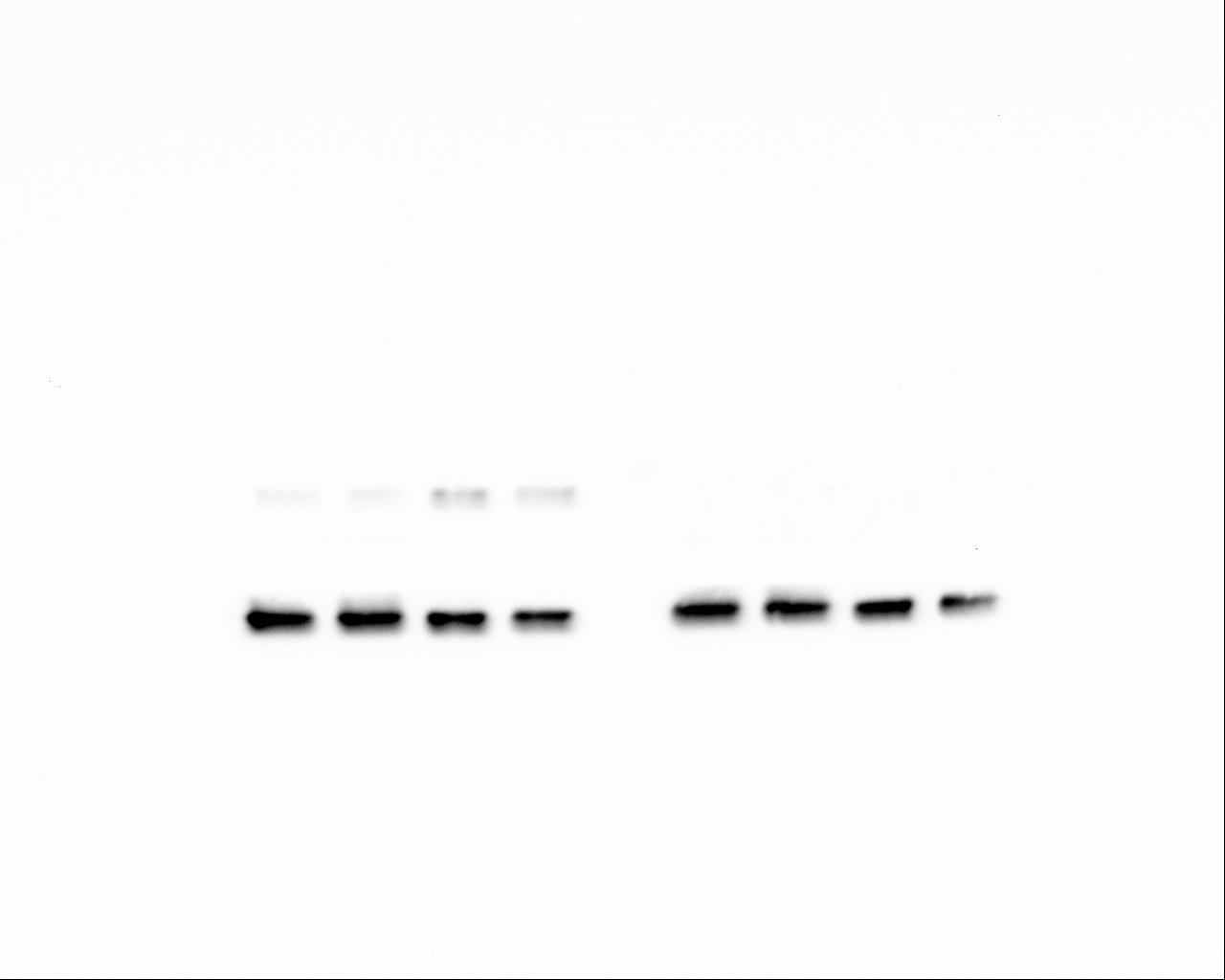

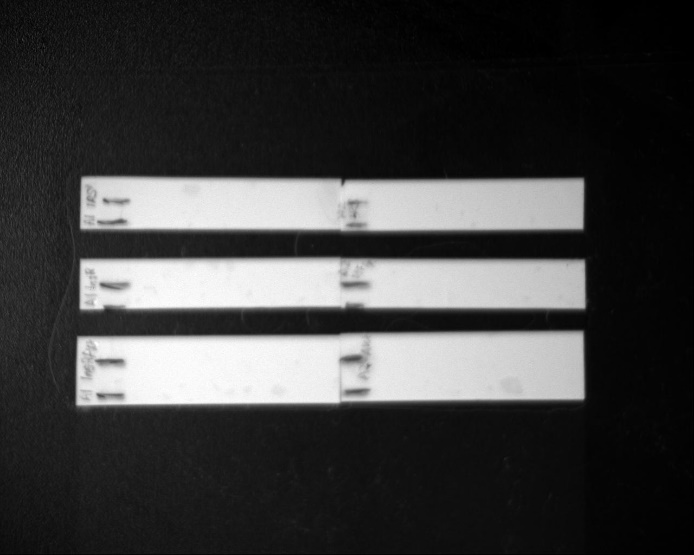


100

70

180

55

130

40

100

70

180

55

130

40

100

70

180

55

130

40

100

70

180

55

130

40

Markers image blots for

Akt and InsR

Akt blot (low exposure)

MW

(kDa)

MW

(kDa)

RIPA - - + +

SMA + + - -

Insulin - + - +


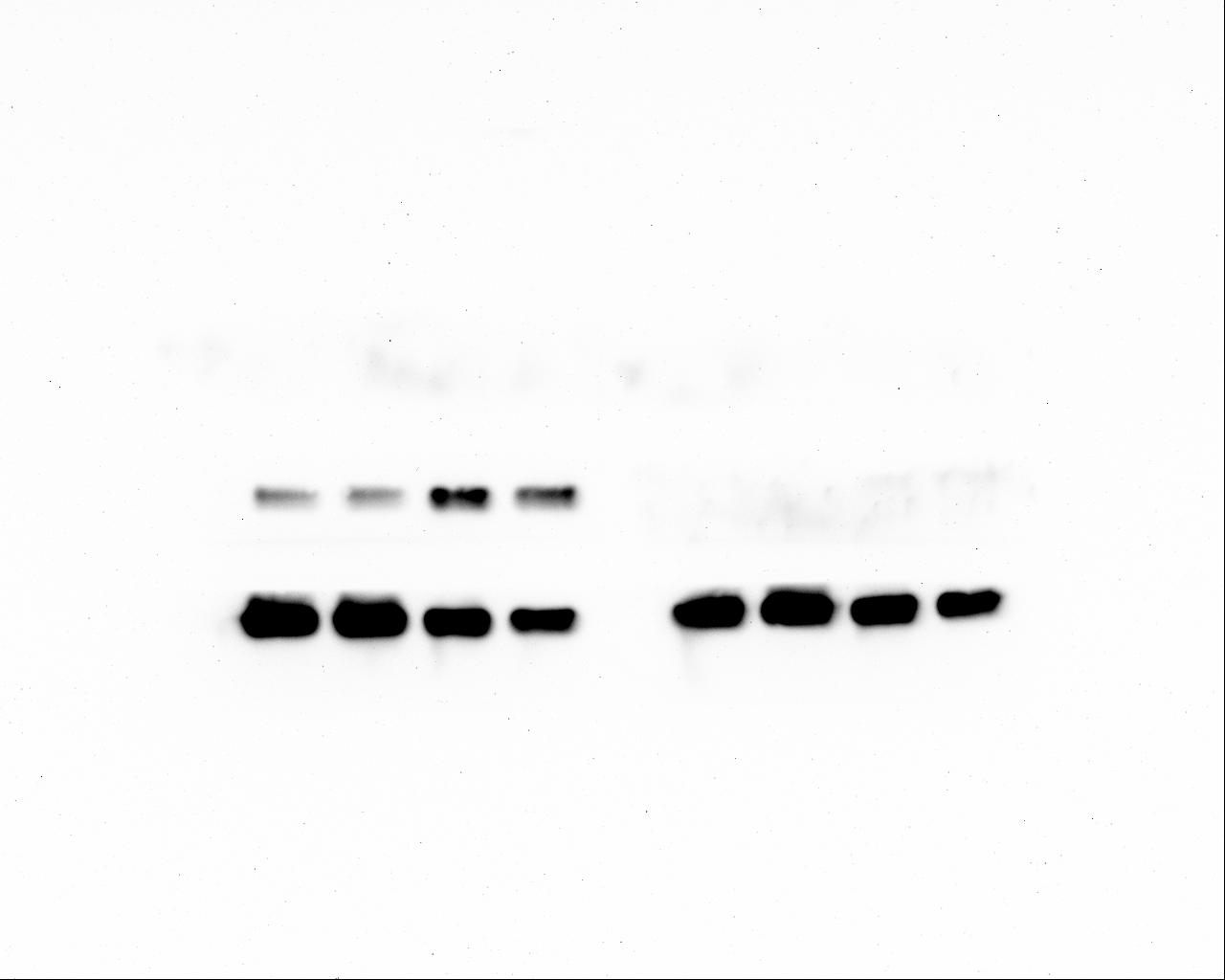


100

70

180

55

130

40

100

70

180

40

130

35

InsR blot (long exposure)

MW

(kDa)

RIPA - - + +

SMA + + - -

Insulin - + - +

RIPA - - + +

SMA + + - -

Insulin - + - +

**Figure 1b (continue)**


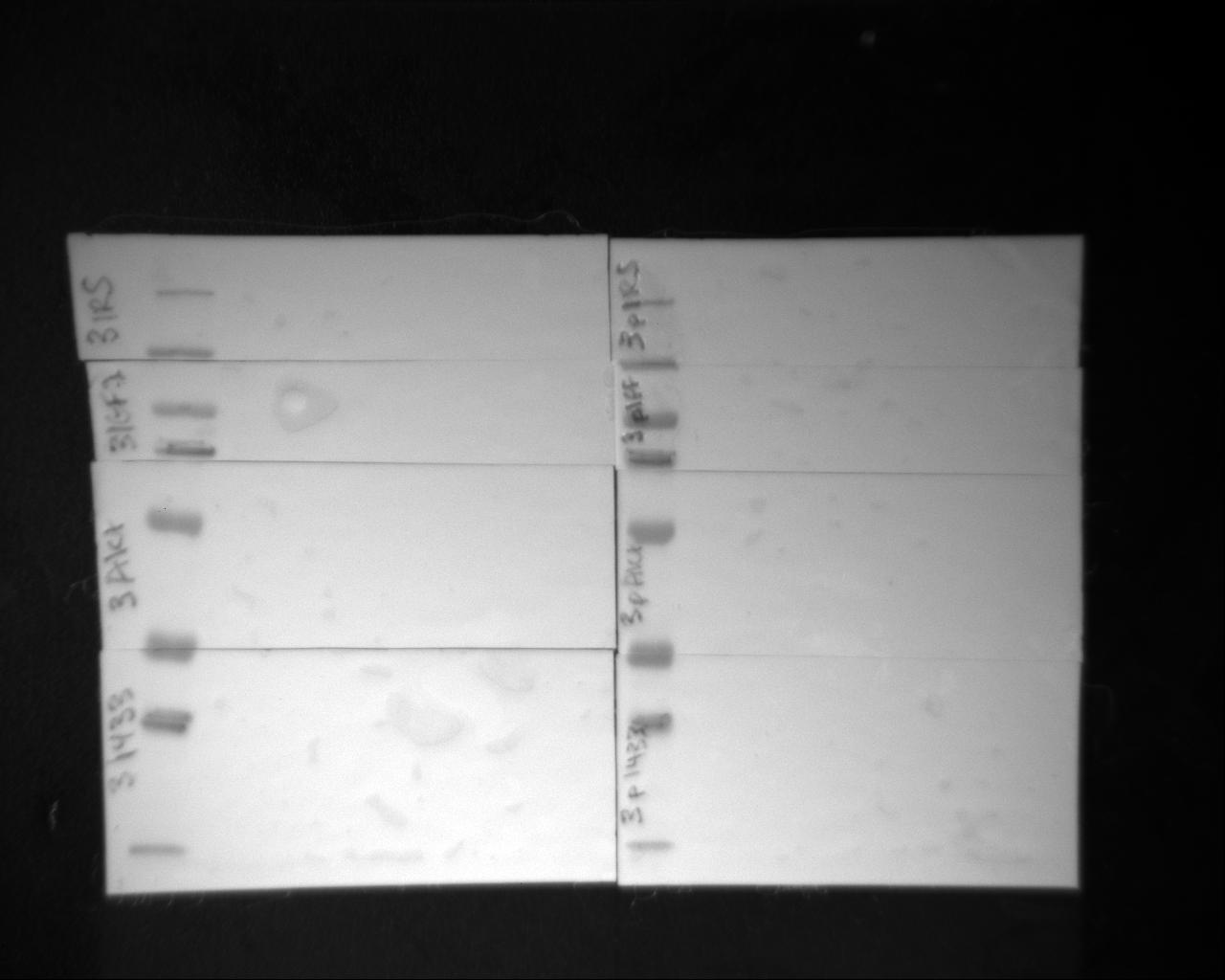


70

55

180

40

130

35

100

25

70

55

180

40

130

35

100

25

Markers image for blot for IRS1, IGF1, Akt and 14-3-3

MW

(kDa)

RIPA - - + +

SMA + + - -

Insulin - + - +


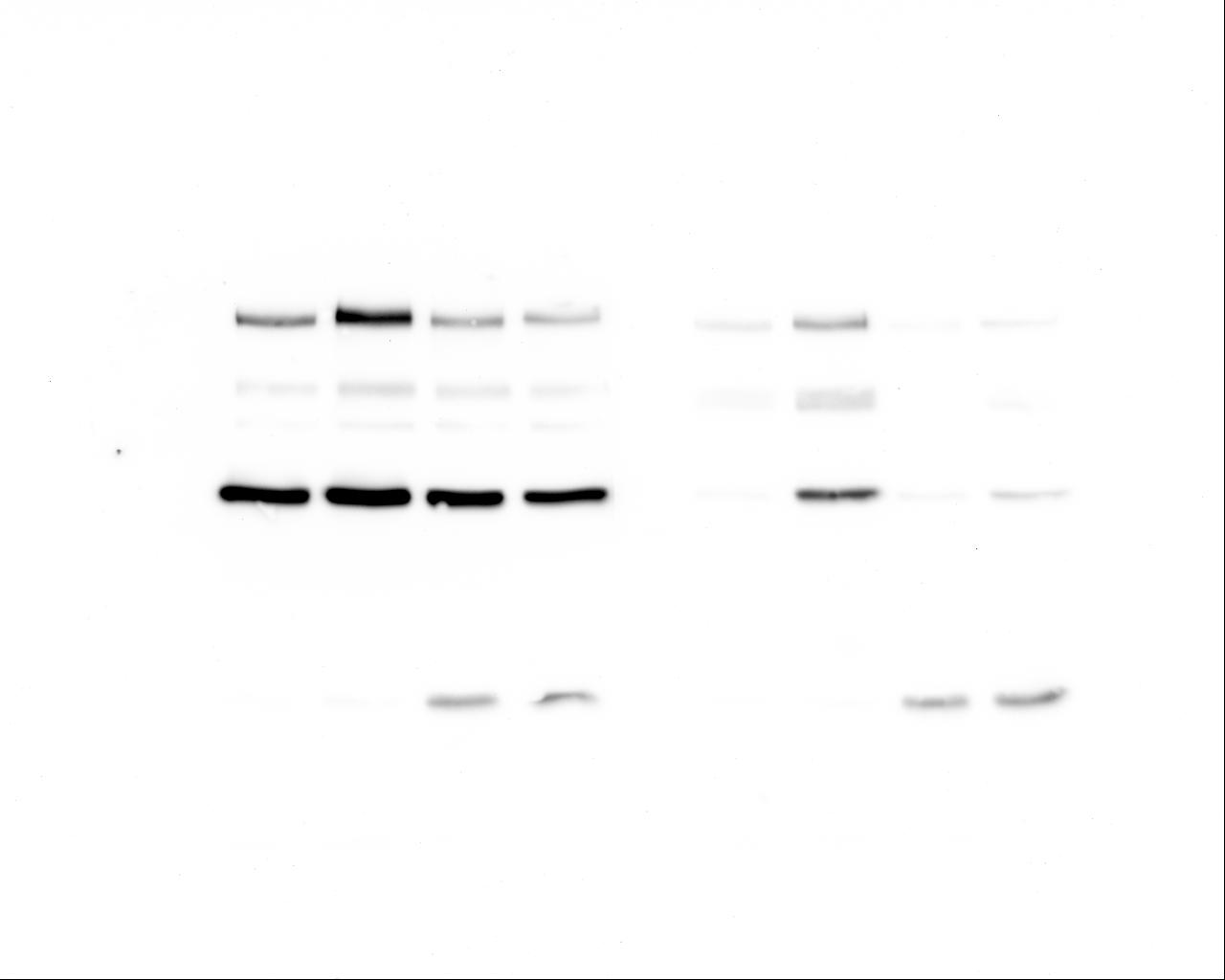


IRS1 and Akt blots (short exposure)

70

55

180

40

130

35

100

25

MW

(kDa)

RIPA - - + +

SMA + + - -

Insulin - + - +

IRS1

Akt


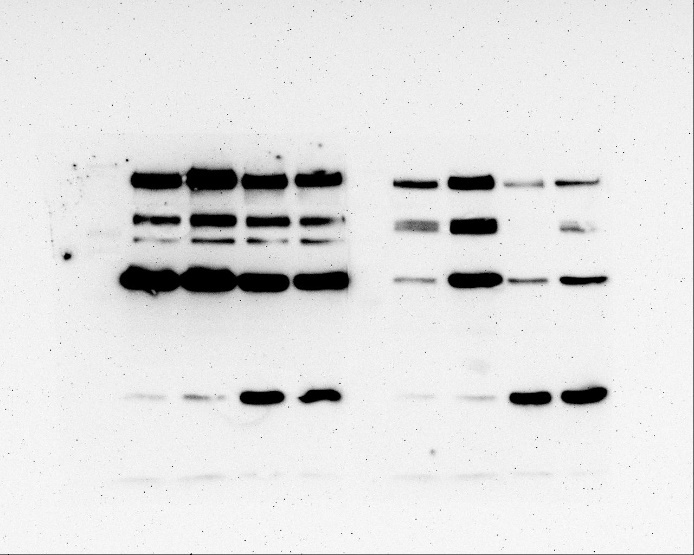


IGF1R and 14-3-3 blots

(long exposure)

70

55

180

40

130

35

100

25

MW

(kDa)

RIPA - - + +

SMA + + - -

Insulin - + - +

IGF1R

14-3-3

**Figure 2a and b**


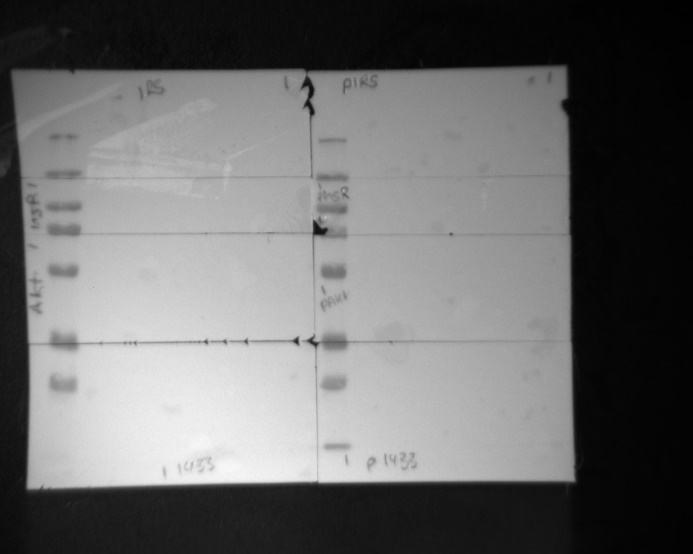


70

55

180

40

130

35

100

Markers image

70

55

180

40

130

35

100

RIPA - - + +

SMA + + - -

Insulin - + - +

- - + +

+ + - -

- + - +


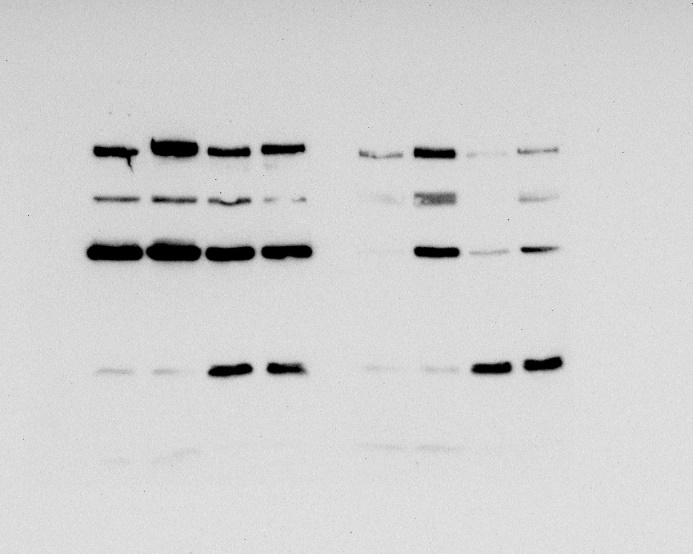


Low exposure blot for IRS1, InsR,

Akt and phospho IRS1

70

55

180

40

130

35

100

RIPA - - + +

SMA + + - -

Insulin - + - +

- - + +

+ + - -

- + - +

pIRS1

IRS1

InsR

Akt


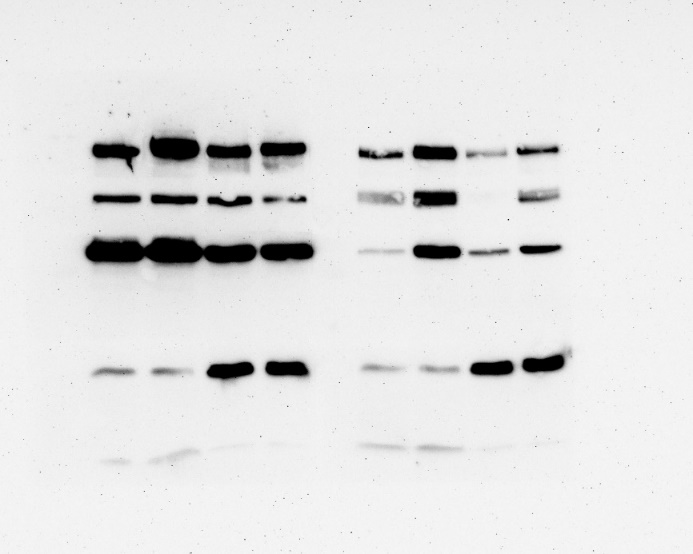


Long exposure blots for

phospho InsR and phospho Akt

70

55

180

40

130

35

100

RIPA - - + +

SMA + + - -

Insulin - + - +

- - + +

+ + - -

- + - +

pInsR

pAkt

MW

(kDa)

MW

(kDa)

MW

(kDa)

**Figure 3**


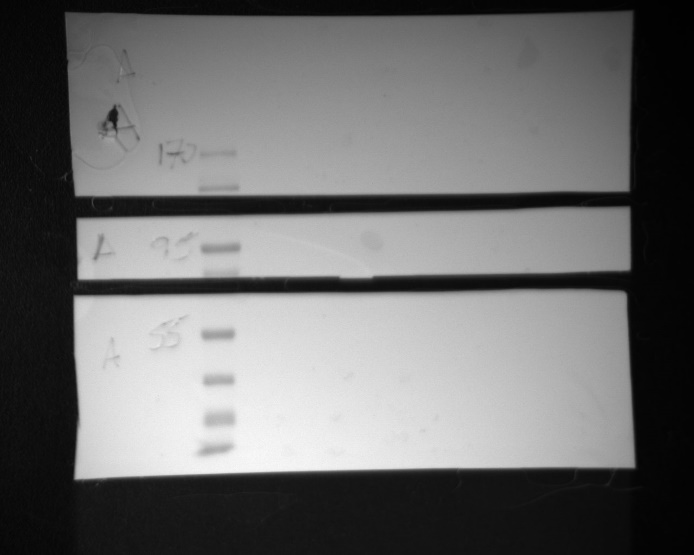


100

70

55

180

40

130

35

25

Markers blot for Akt

(low intensity ECL reagent)


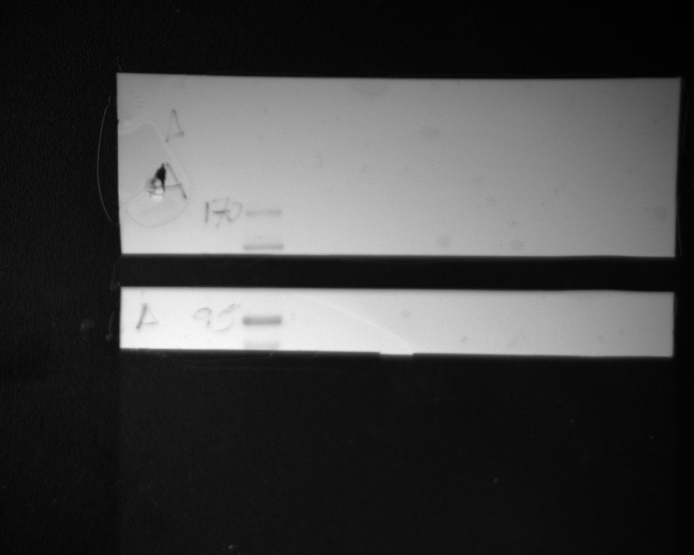


100

180

130

70

Markers blot for InsR and PDGFR

(high intensity ECL reagent)


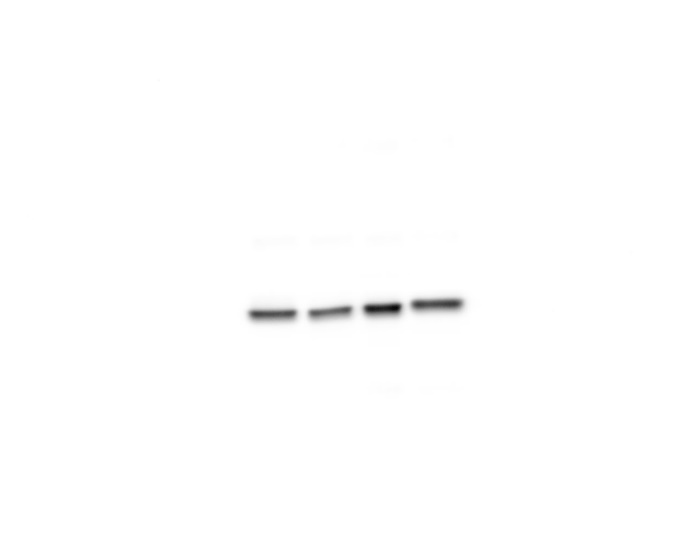


100

70

55

180

40

130

35

25

Total Akt Blot

SMA extracted

RIPA extracted

-ve control

+ve control


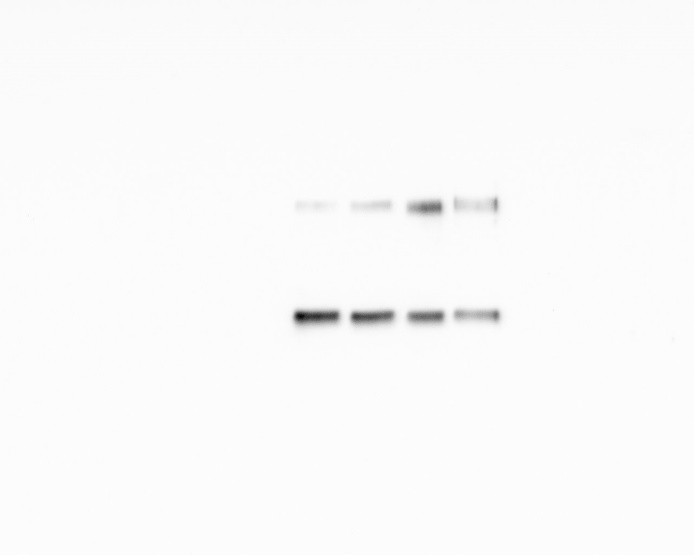


100

180

130

70

Total InsR blot (short exposure)

SMA extracted

RIPA extracted

-ve control

+ve control


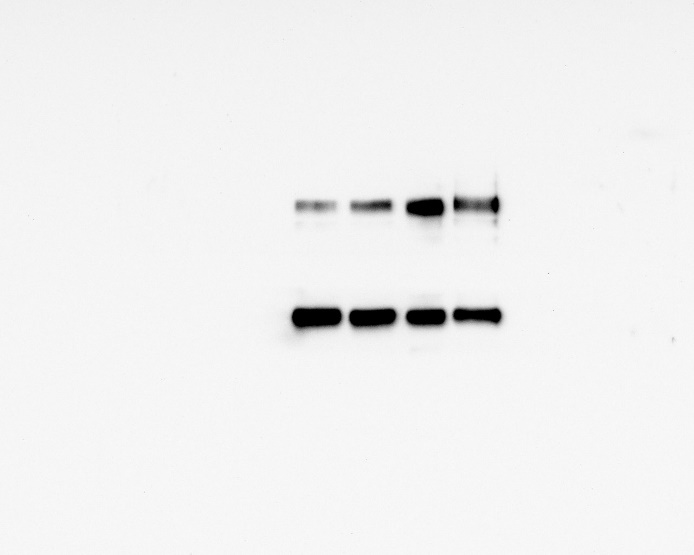


100

180

130

70

Total PDGFRα (long exposure)

SMA extracted

RIPA extracted

-ve control

+ve control

MW

(kDa)

MW

(kDa)

MW

(kDa)

MW

(kDa)

MW

(kDa)

**Figure 3 (continue)**


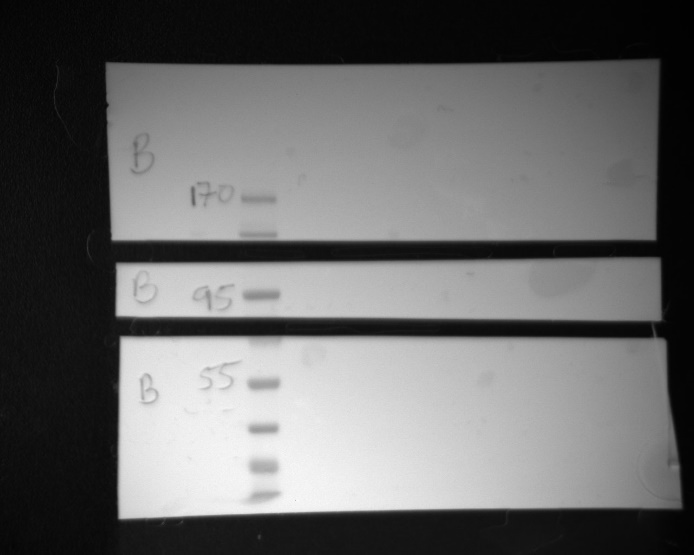


100

70

55

180

40

130

35

25

Markers image for pAkt (low intensity ECL reagent)


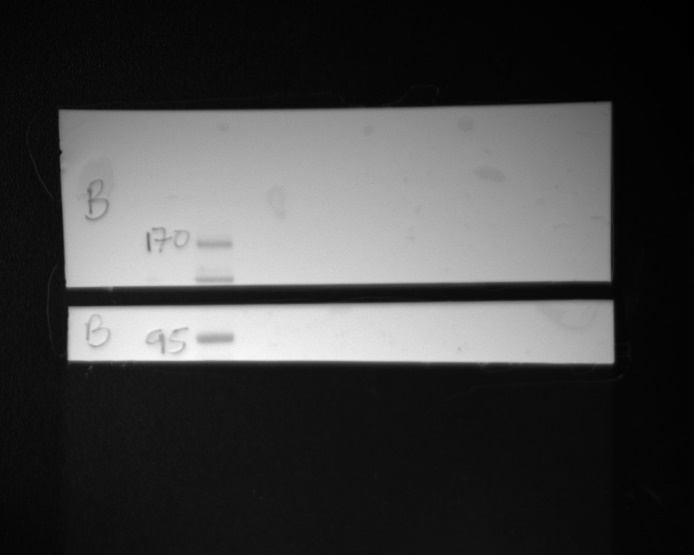


100

180

130

Markers image for pInsR and pPDGFR ) (high intensity ECL reagent)


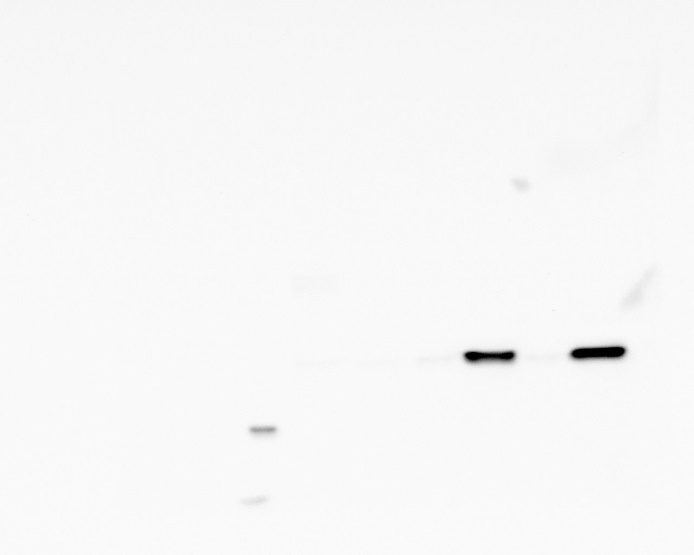


100

70

55

180

40

130

35

25

phospho Akt

SMA extracted

RIPA extracted

-ve control

+ve control


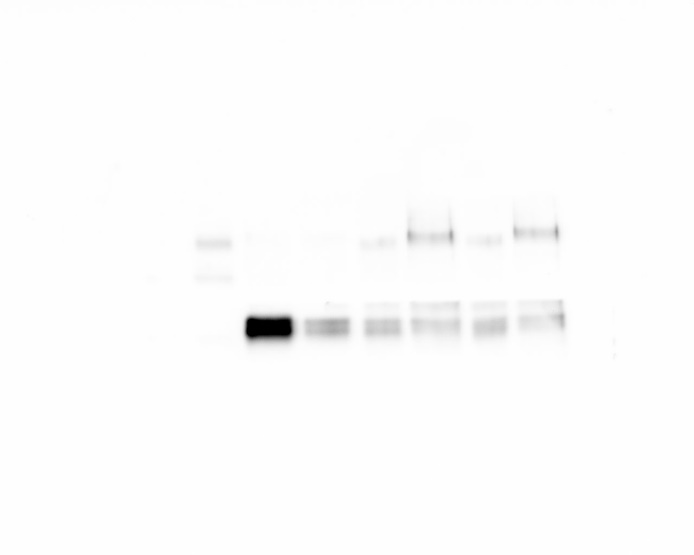


100

180

130

phospho InsR blot (short exposure)

SMA extracted

RIPA extracted

-ve control

+ve control


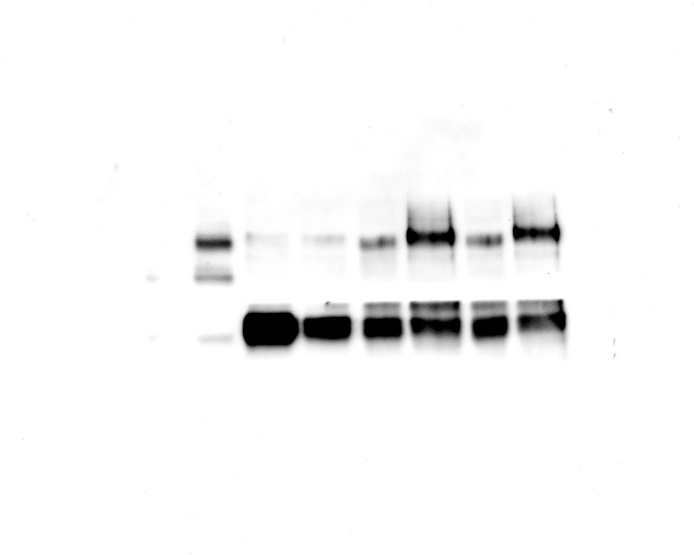


100

180

130

phospho PDGFR (long exposure)

SMA extracted

RIPA extracted

-ve control

+ve control

MW

(kDa)

MW

(kDa)

MW

(kDa)

MW

(kDa)

MW

(kDa)

MW

(kDa)

MW

(kDa)

**Figure 4a**


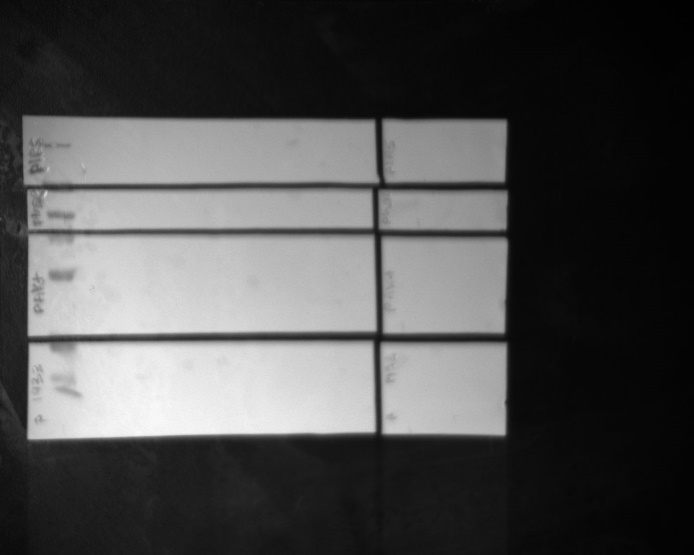


Markers image Blot for pIRS1, pInsR and pAkt

100

70

55

180

40

130

35

100

70

55

180

40

130

35


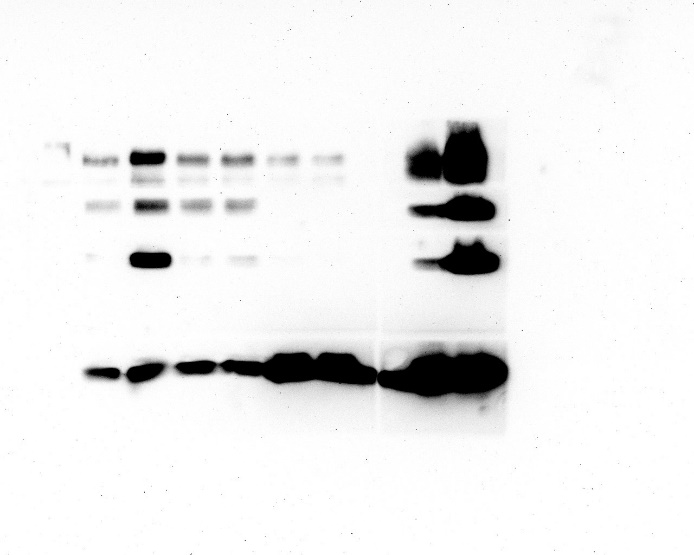


*In vivo*

*In vitro*

RIPA - - - - + +

SMA + + + + - -

Insulin - + - + - +

pIRS1

pInsR

pAkt

MW

(kDa)

MW

(kDa)

Markers image Blots for total Akt (short exposure)


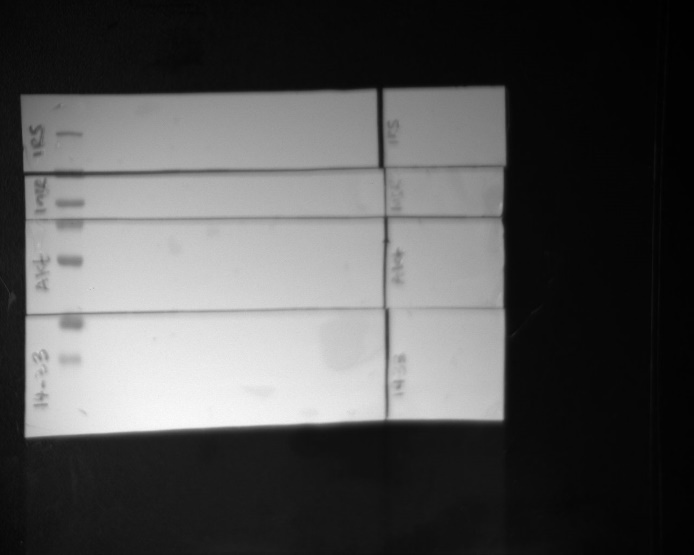


100

70

55

180

40

130

35


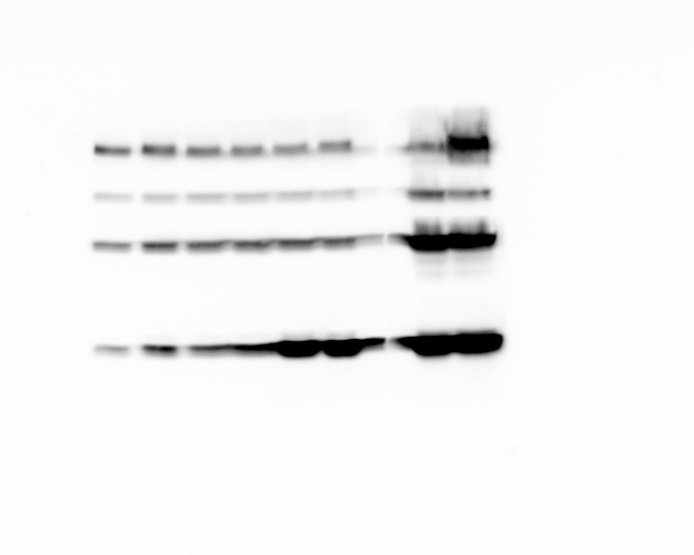


100

70

55

180

40

130

35

RIPA - - - - + +

SMA + + + + - -

Insulin - + - + - +

*In vivo*

*In vitro*

Blots for total IRS1, InsR

(long exposure)


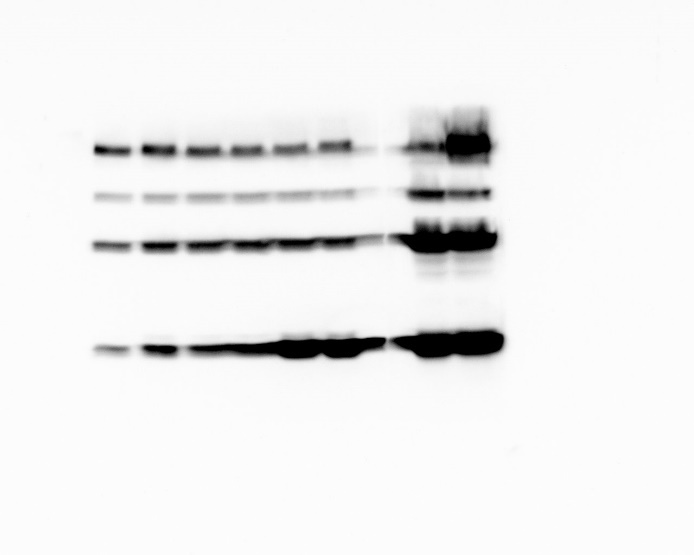


100

70

55

180

40

130

35

RIPA - - - - + +

SMA + + + + - -

Insulin - + - + - +

*In vivo*

*In vitro*

**Figure 4b**

MW

(kDa)

MW

(kDa)

MW

(kDa)

**Supplementary Fig S2**

Markers for Akt, IRS1 and InsR Blots for Akt and total IRS1

(low exposure time)

Markers for pIRS1, pInsR and pAkt Blots for pIRS1 pInsR and pAkt


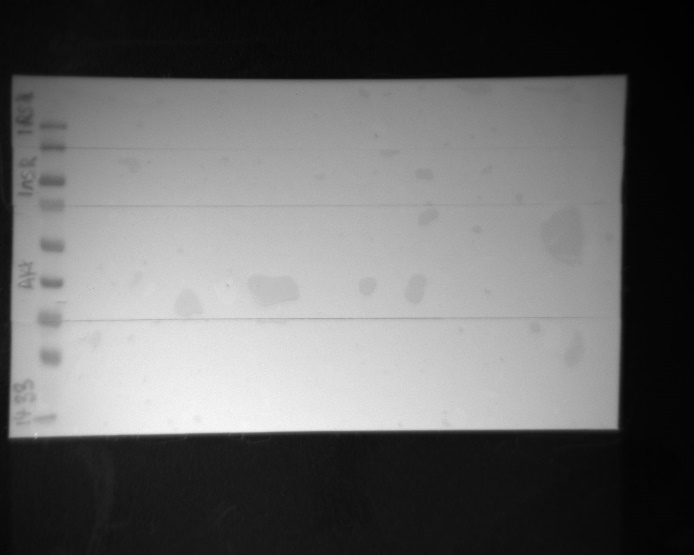


70

55

180

40

130

35

100

15

25


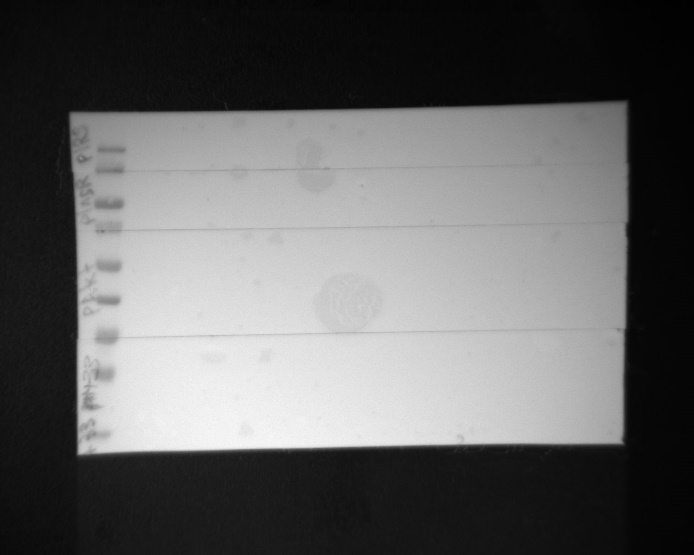


70

55

180

40

130

35

100

15

25


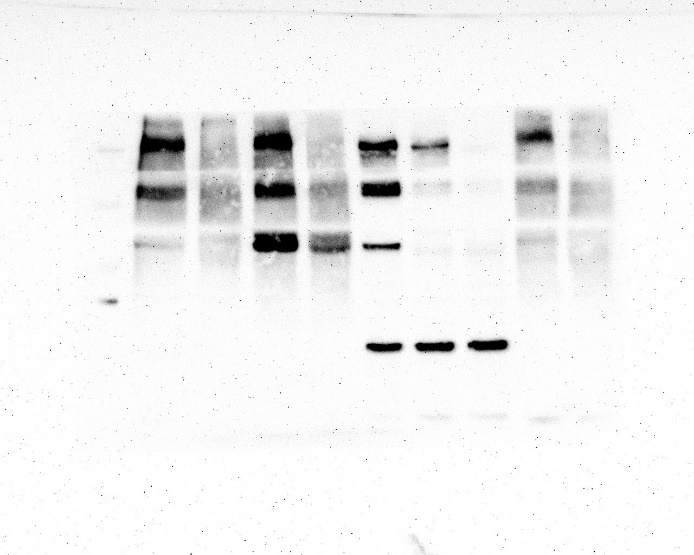


70

55

180

40

130

35

100

15

25

SMA + + + +

CIP - + - +

Insulin - - + +


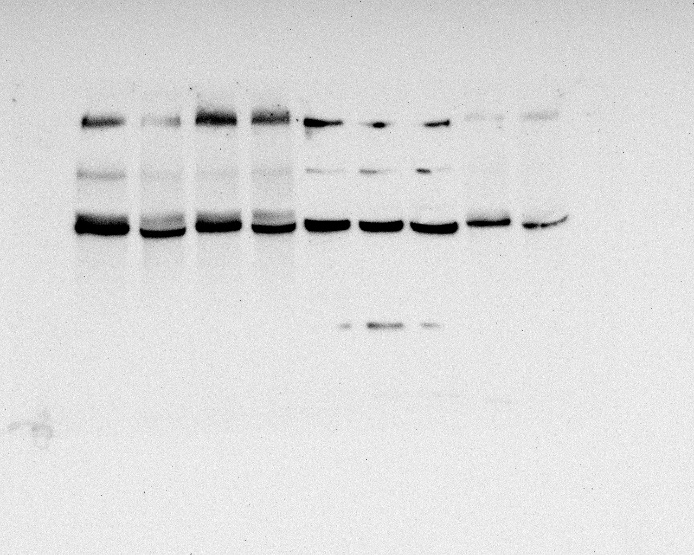


70

55

180

40

130

35

100

15

25

SMA + + + +

CIP - + - +

Insulin - - + +

IRS1

Akt


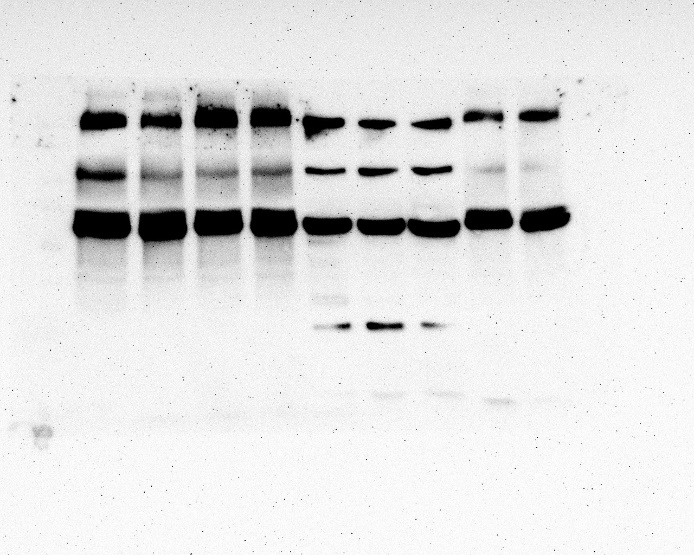


Blot for Total InsR

(long exposure time)

70

55

180

40

130

35

100

15

25

SMA + + + +

CIP - + - +

Insulin - - + +

InsR

pIRS1

pInsR

pAkt

MW

(kDa)

MW

(kDa)

MW

(kDa)

MW

(kDa)

MW

(kDa)

**Supplementary** **Fig S3**


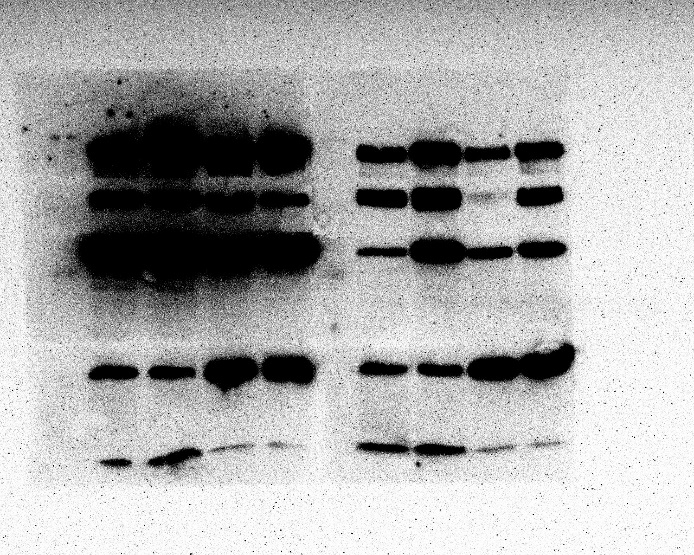

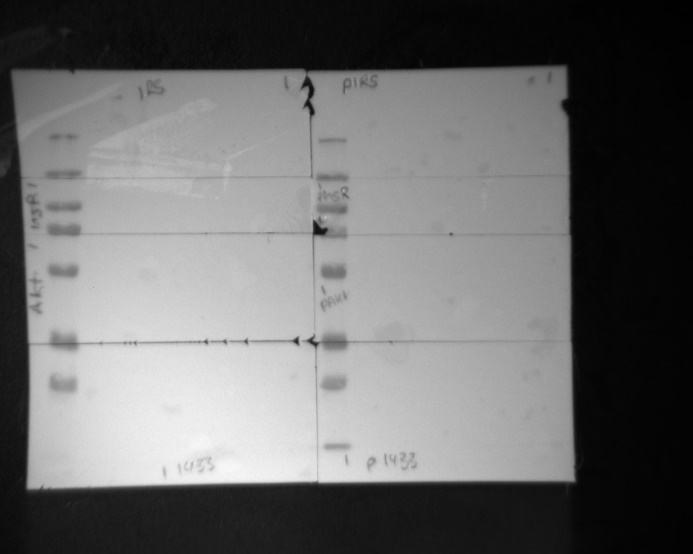


70

55

180

40

130

35

100

25

Markers image

Overexposed blot for phospho InsR

Supplementary Figure S3

70

55

180

40

130

35

100

25

RIPA - - + +

SMA + + - -

Insulin - + - +

MW

(kDa)

MW

(kDa)

**Supplementary Fig S4**

Phospho PDGFRα, phospho InsR and total Akt blots on membrane (1)


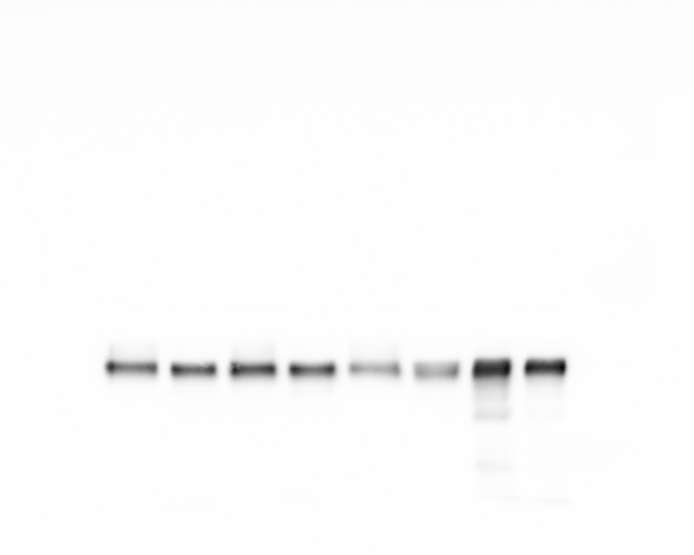

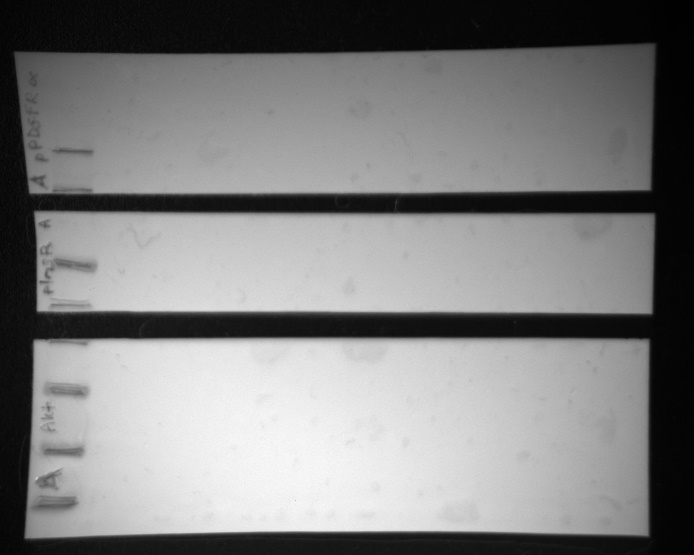


Markers Akt blot total Akt blot

(low intensity ECL reagent)

100

70

180

55

130

40

35

100

70

180

55

130

40

35

Exp1 Exp2 Exp3 Control

SMA + - + - + -

RIPA - + - + - +


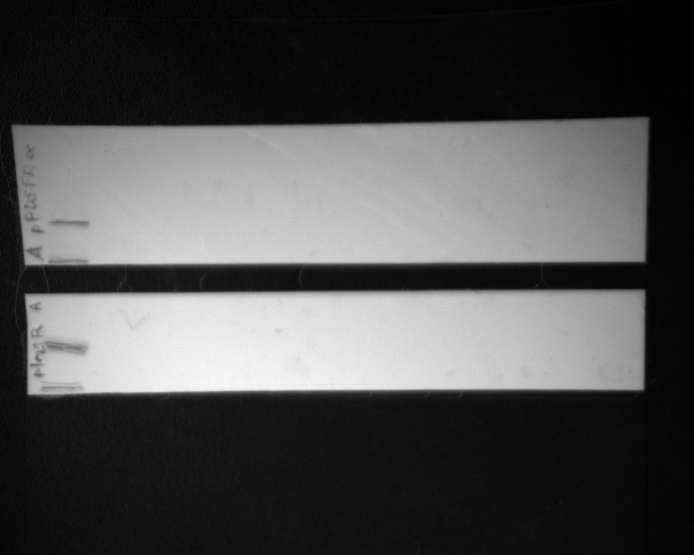

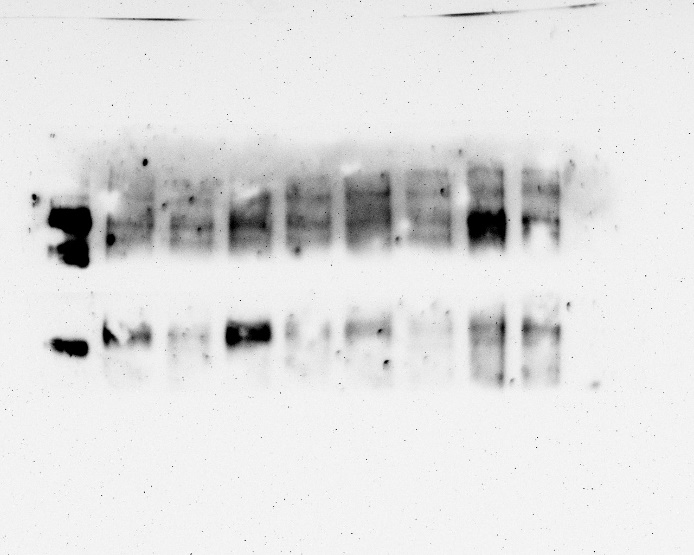


Markers pPDGFRα, pInsR Phospho PDGFRα, phospho InsR blots

(high intensity ECL reagent)

70

130

100

180

70

130

100

180

Exp1 Exp2 Exp3 Control

SMA + - + - + -

RIPA - + - + - +

pPDGFRα

pInsR

MW

(kDa)

MW

(kDa)

MW

(kDa)

MW

(kDa)

**Supplementary** **Fig S4**

Total PDGFRα, InsR and Akt blots on membrane (2)

Markers (low intensity ECL reagent) Total Akt


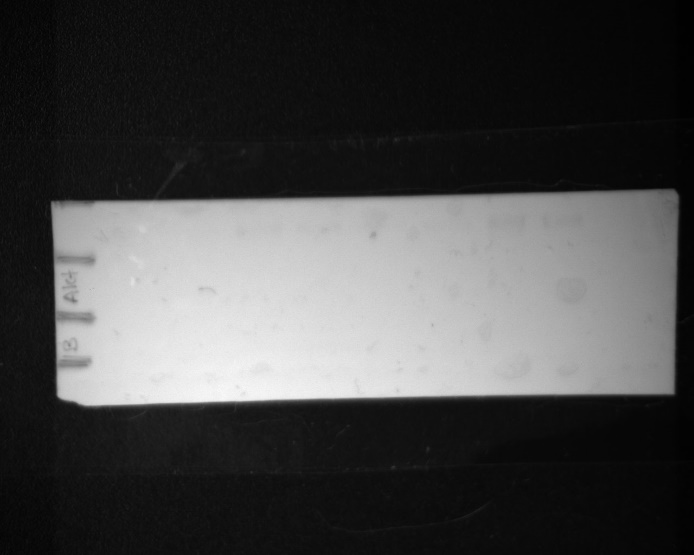


70

55

40

35

Markers (high intensity ECL reagent)

Total InsR (low exposure) Total PDGFRα (long exposure time)


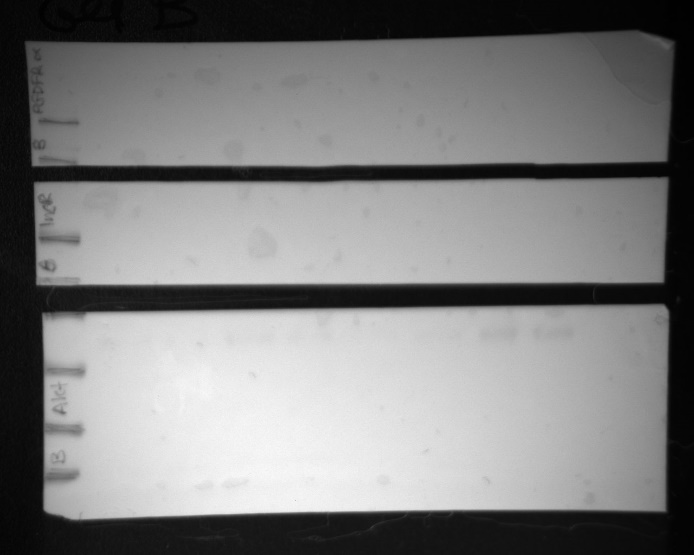


130

100

70

55

180

40

35


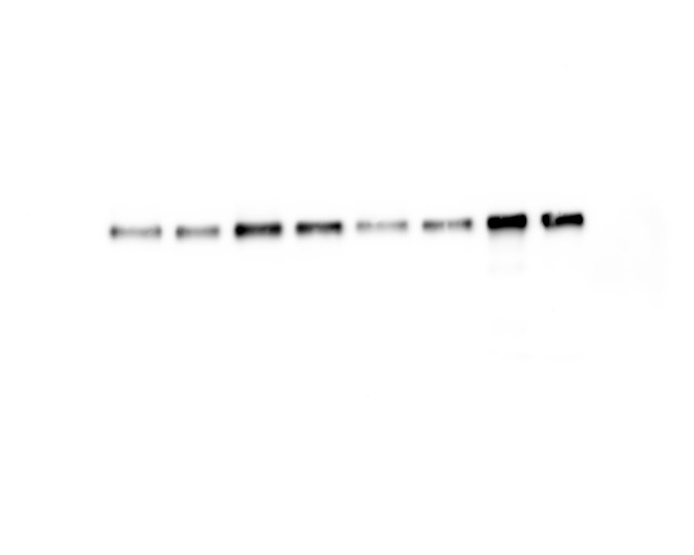


70

55

40

35

Exp1 Exp2 Exp3 Control

SMA + - + - + -

RIPA - + - + - +


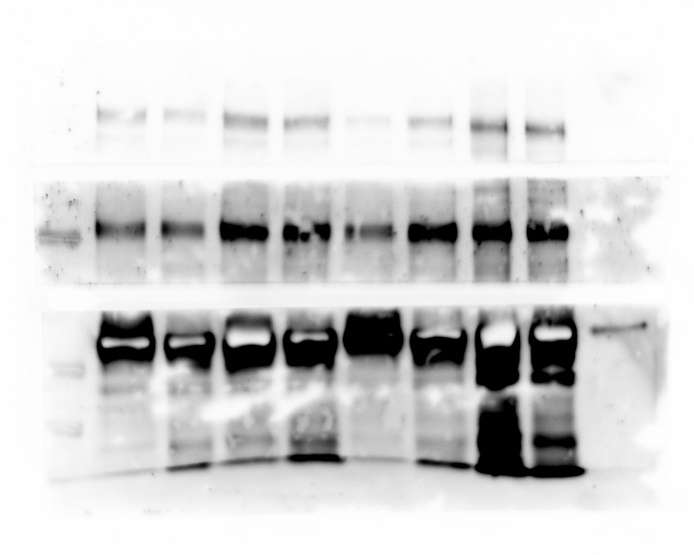


130

100

70

55

180

40

35

Exp1 Exp2 Exp3 Control

SMA + - + - + -

RIPA - + - + - +


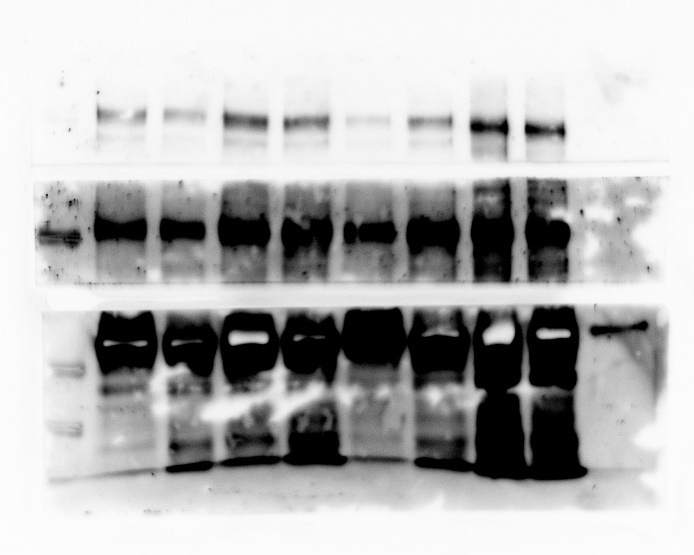


130

100

70

55

180

40

35

Exp1 Exp2 Exp3 Control

SMA + - + - + -

RIPA - + - + - +

MW

(kDa)

MW

(kDa)

MW

(kDa)

MW

(kDa)

MW

(kDa)
